# Dietary Overlap of Sympatric Polyphagous Alpine Grasshoppers Includes Invasive Plant Species

**DOI:** 10.1002/ece3.73576

**Published:** 2026-04-29

**Authors:** Mari Nakano, Steven A. Trewick, Richard N. Watson, Mary Morgan‐Richards

**Affiliations:** ^1^ Wildlife & Ecology, SFTNS Massey University Palmerston North New Zealand; ^2^ Lincoln College, University of Canterbury Christchurch New Zealand

**Keywords:** Acrididae, *Chionochloa* snow tussock, chloroplast *trn*L, DNA metabarcoding, grasshopper diet, invasive plant species, mandible, micro‐histology, New Zealand

## Abstract

Understanding what herbivores eat can provide important information about competitive interaction among sympatric species and the potential for selective feeding to shape plant communities. The flightless alpine grasshoppers *Sigaus australis,* *S. nitidus* and *S. nivalis* are sympatric and abundant in the mountains of South Island, Aotearoa New Zealand. We investigated whether their diets differ among locations, species and sexes, and whether the plants that they ate had changed over 50 years. Mandible morphology of the grasshopper species was compared to infer diet adaptation. Their diet was analysed using gut samples collected from 1969 to 1971 and 2021 to 2023. Despite differences in mandibles suggesting potential adaptation to food plants of different sizes and toughness, we found greater diet differences among locations than among grasshopper species or between sexes. All three *Sigaus* grasshoppers consume a wide range of plants. Considered as a guild, we found the diet of these grasshoppers was influenced by local environment and season, but that they did not feed at random. Shrubs and herbs including *Gaultheria* and *Lobelia* were favoured, but the abundant *Chionochloa* grasses were avoided. Despite narrow seasonality, fruits and flowers were also important dietary components. Invasive plants including *Hieracium, Pilosella* and *Taraxacum* that were rare or absent in the diet 50 years earlier had been eaten by 92% of the grasshoppers sampled in 2021–2023. By selectively feeding on soft groundcover and invasive plants, *Sigaus* grasshoppers may act as ecosystem architects in the alpine plant communities that continue to be dominated by snow tussock.

## Graphical abstract

The diets of three sympatric grasshopper species were compared using mandible morphological analysis, microhistological examination, and DNA metabarcoding of gut contents. Although mandible morphology differed across species and sexes, dietary divergence was not evident. Instead, diet appeared to be more strongly influenced by local and temporal variation in plant availability. Moreover, we observed dietary shifts over time, with recent samples including invasive weeds that were absent from specimens collected 50 years ago.

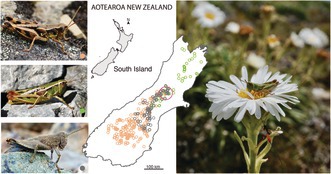

## 1 Introduction

Grasshoppers are ecologically influential herbivores in many open habitats where they influence the composition and structure of plant communities (e.g., Chen et al. 2024; Grime 2001; Laws et al. 2018; Le Gall et al. 2019; Torrusio et al. 2002). Though individually small, they are often numerically abundant, and the group is taxonomically diverse displaying varying degrees of dietary specialisation (Chen et al. 2024; Joern 1979a, 1979b; Ren et al. 2024). Grasslands, where seasonal climate variation in the form of drought or cold temperature restricts growth of woody plants (Petermann and Buzhdygan 2021; Risser 1988), constitute about 40% of terrestrial habitat and tend to have high plant diversity over small spatial scales (Petermann and Buzhdygan 2021). A combination of top‐down (Jia et al. 2018) and bottom‐up processes (Bröcher et al. 2024; Joshi et al. 2008; Scherber et al. 2010) yield taxonomic diversity and ecological complexity. Herbivory by grasshoppers and other invertebrates is an ecologically influential process, as their life histories align with seasonal cycles of climate and plant growth (Loranger et al. 2014; Welti et al. 2019), shaping evolutionary ecology (Joern 1979a; Olff and Ritchie 1998).

Invertebrate herbivores include specialists that target particular plant species but most are polyphagous feeders that utilise segments of the available flora depending on growth‐form or nutritional traits (Bröcher et al. 2024; Ibanez, Lavorel, et al. 2013; Ibanez, Manneville, et al. 2013; Joern 1979a, 1979b). Among grasshoppers that have been studied, some species appear to feed either on herbs or grasses, whereas others use both (Ibanez, Lavorel, et al. 2013; Joern 1979a; McClenaghan et al. 2015). As well as selecting among available plant species, emphasis on particular plant parts including flowers and seeds can directly impact plant reproduction (White and Watson 1972). As a result, the functional composition of orthopteran communities is likely to influence plant community composition (Laws et al. 2018).

Insect mouthparts are specialised for feeding on different fractions of a flora (Krenn 2019). Short‐horned grasshoppers are biting and chewing insects feeding primarily on plant tissue (Clissold 2007; Krenn 2019) and examination of their mandibles reveals two broad morphotypes. Species that eat non‐woody herbs and shrubs have sharp incisors and molars, while grass‐eating grasshoppers have blunt incisors and molars (Gangwere 1965; Isely 1944). These mandibular forms probably relate to physical differences in leaf shape and toughness (Clissold 2007; Ibanez, Lavorel, et al. 2013). As in many insects, grasshopper mandibles are sclerotised with melanin and/or metals (e.g., zinc, manganese, or iron) (Krenn 2019) that provides resistance to wear. The extent of hardening may reflect use of different plant types.

Quantifying the diet of small herbivores is challenging and typically a combination of approaches is most revealing. Direct observations can provide compelling insight about behaviour and diet (e.g., Ibanez, Lavorel, et al. 2013; Ibanez, Manneville, et al. 2013; Mallott et al. 2018), but only represent brief periods of feeding. Less direct approaches can provide a better overview of dietary range by examining the plant materials of gut contents or frass via microhistological epidermal analysis (e.g., Joern 1979b; Johnson et al. 1983; Soininen et al. 2009; Trewick 1996) and molecular techniques (Heise et al. 2015; Jurado‐Rivera et al. 2009; Mallott et al. 2018; McClenaghan et al. 2015; Prewer et al. 2023; Soininen et al. 2009; Welti et al. 2019).

In New Zealand, natural open habitat is almost entirely associated with the alpine zone where a distinctive ecosystem has evolved featuring endemic plants and animals (Mark et al. 2000; Raven 1973). Grasshoppers in the Swiss Alps eat up to 30% of above‐ground plant biomass (Blumer and Diemer 1996), and in the New Zealand Southern Alps, similar grazing pressure appears to be exerted by grasshoppers eating low‐abundance ground cover species (Batcheler 1967, White 1974a, 1974b, 1975). Grasshoppers of the endemic *Sigaus* radiation (Trewick et al. 2023) represent the majority of invertebrate biomass in New Zealand alpine areas (Chinn and Chinn 2020; White 1974a) and so may significantly influence vegetation characteristics and composition. Their habitat includes fellfield, scree/rock, tussock grasslands, scrub and herbfields with cushion plants (Bigelow 1967; Hudson 1970) and more than 500 species of ferns, rushes/sedges, grasses, herbs, and shrubs grow in these areas (Heenan and McGlone 2013; Mark et al. 2000; McGlone et al. 2001). This alpine vegetation is dominated by coarse‐leaved *Chionochloa* tussock grasses. Most areas are now under pressure from a range of human‐mediated changes including invasive plant species (Day et al. 2023; Halloy and Mark 2003; Steer and Norton 2013) with for example, native *Festuca* and short tussock *Poa* species in dry low elevation grasslands (< 1200 m asl) of South Island being rapidly replaced by invasive hawkweeds *Pilosella officinarum*, *Hieracium lepidulum*, and *H. praealtum* (Day and Buckley 2011; French 2021; Steer and Norton 2013; Wiser et al. 2016). Among *Hieracium* species, *H. lepidulum* appears to be the most abundant at higher elevations and its invasion of areas dominated by tall tussock (*Chionochloa*) has occurred in the last 25 years (Day and Buckley 2011). Invasive plants including heather *Calluna vulgaris* , wilding conifers *Pinus* species, sheep's sorrel *Rumex acetosella* , clover *Trifolium* species, catsear *Hypochaeris radicata* , and grass *Agrostis capillaris* are also now commonly seen in New Zealand high country (Moyle and Deslippe 2024; Owen 1998; Wiser et al. 2016). The impact of invasive species on herbivorous insects in New Zealand has not been directly studied but is implicated in the range change of some endemic grasshoppers (Sivyer et al. 2018).

**FIGURE 1:**
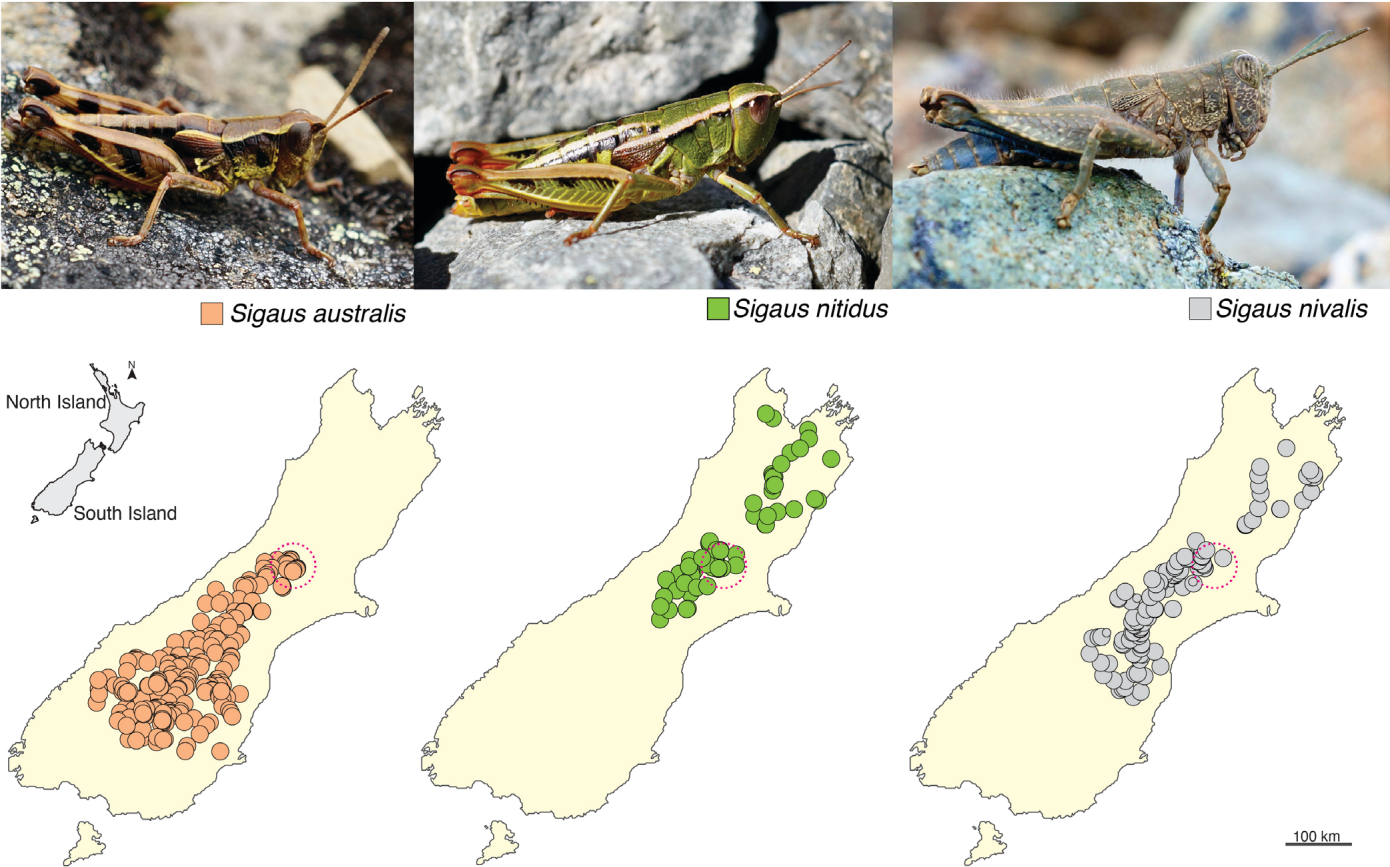
Three species of the endemic New Zealand *Sigaus* grasshopper radiation and their mapped distributions (Trewick et al. 2023). The area encompassing four sampling sites in the present study is shown by dashed pink circle, where the three *Sigaus* species also co‐occur. Photos by Steve Trewick.

We investigated feeding overlap among species of *Sigaus* (Hutton 1897) grasshoppers, where they are sympatric in alpine vegetation of the Southern Alps of New Zealand (Figure 1), comparing mandible morphology and gut contents of adults. Sympatry could drive selection for resource partitioning resulting in divergence of mandibles and food‐plant choices among grasshopper species (Brown and Wilson 1956). As females are significantly larger than males in all *Sigaus* species (Meza‐Joya et al. 2022; Bigelow 1967), sexes might also show dietary differentiation. We then analysed the diets of *Sigaus* species as an ecological guild to test the prediction that their foraging reflects food plant availability rather than taxon‐specific selection by comparing vegetation and ingestion frequencies. We compared *Sigaus* gut content data collected in 1968–1970 with sampling 50 years later in 2021–2023 for evidence of dietary shifts that might reflect vegetation changes including spread of invasive weeds in the alpine zone.

## 2 Materials and Methods

### 2.1 Study Area and Sampling

Sampling was conducted at four mountains in the Canterbury region of the Southern Alps—Kā Tiritiri o te Moana, New Zealand (Figure 1), where three *Sigaus* grasshopper species are sympatric: Mount Hutt (−43.5118, 171.5492), Fox Peak (−43.8530, 170.8077), Foggy Peak (−43.294107,171.744770), Craigieburn Range (−43.125750, 171.686239). Adult grasshoppers were collected in alpine habitat 1340–1700 m above sea level with authority from the New Zealand Department of Conservation (permit numbers: 49878‐RES; 97,397‐FLO). At each location, we collected specimens of all three species within 100 m of each other. Sampling took place during the day in the summer (December to April) when grasshoppers were active. During the New Zealand winter (April to November), access to high elevation sites was not possible due to freezing conditions and snow cover, and grasshoppers were inactive among vegetation and rocks.

**FIGURE 2:**
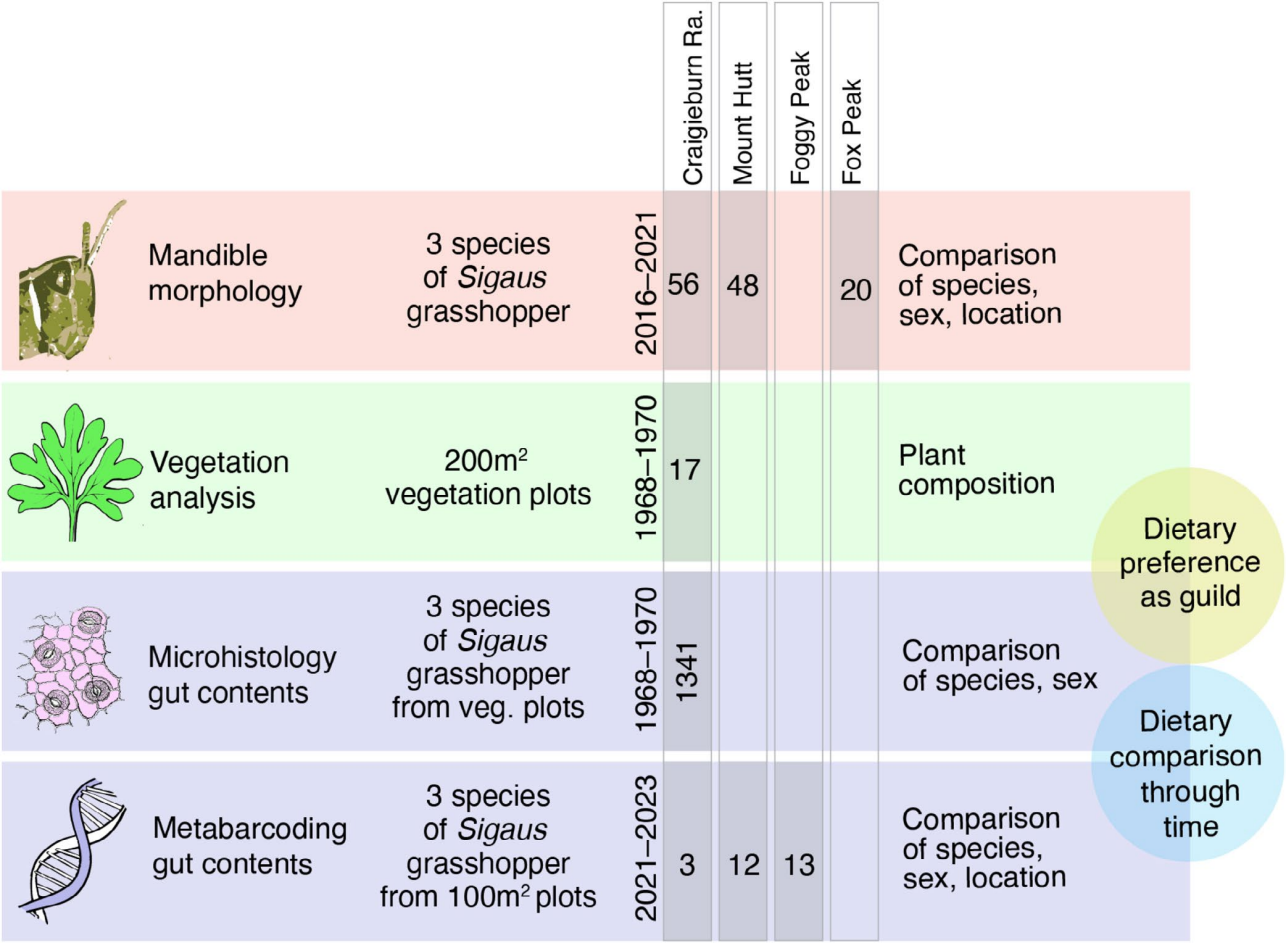
Sampling strategy for feeding and diet of sympatric grasshoppers in central South Island, New Zealand. Location, timing and scale of sampling are indicated for four mountain sites.

Three phases of sampling were used: 1968–1970 vegetation plots for plant composition and grasshopper diet, 2016–2021 for morphological comparison of mandibles, and 2021–2023 for grasshopper diet (Figure 2).

### 2.2 Mandible Morphology

**FIGURE 3:**
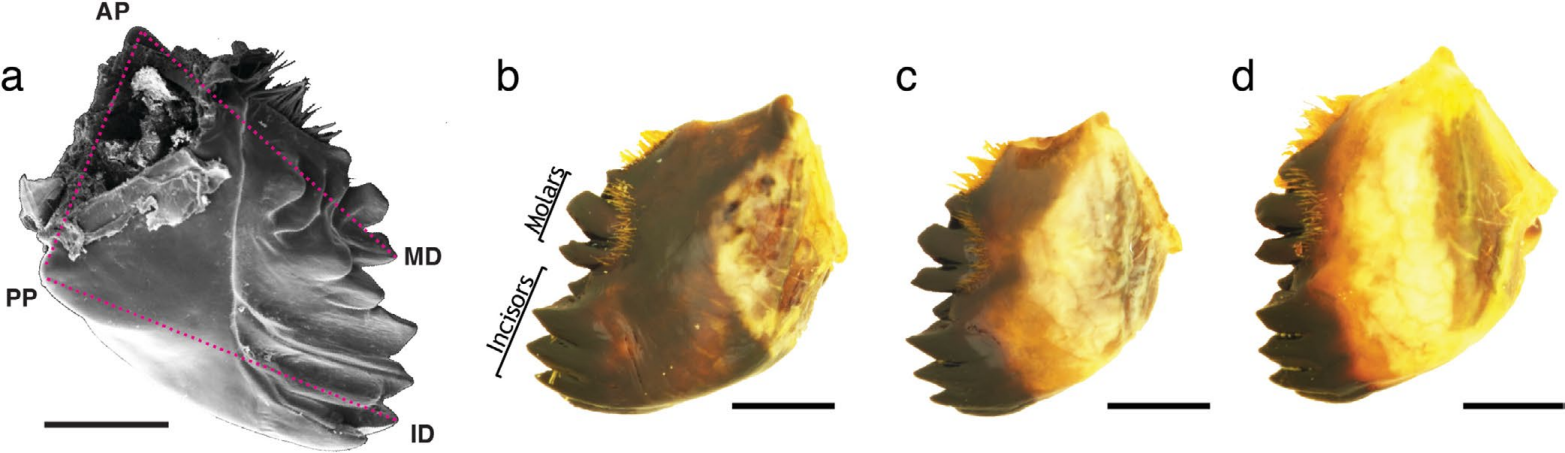
Mandibles of *Sigaus* grasshoppers have different dimensions and proportions of melanisation that might be adaptations to different food plant species or tissues. (a) Posterior view of the left mandible from female *S. nivalis* grasshopper. ID = incisor dents, M = molar dents, PP = posterior articular process, AP = anterior articular process. Linear measurements (pink dashed lines) were made of incisor length (PP‐ID), hinge length (PP‐AP) and molar length (AP‐MD). Anterior surfaces of left mandible showing melanisation on female (b) *S. australis* , (c) *S. nitidus* , (d) *S. nivalis* . Scale bar = 1 mm.

Adult *Sigaus australis* (18 female, 16 male), *S. nitidus* (22 female, 24 male), and *S. nivalis* (20 female and 24 male) were collected from three locations where they are sympatric (Figure 2; Table S1). We examined relative size and degree of sclerotisation of grasshopper mandibles (Figure 3), which are traits linked to feeding strategy (Gangwere 1965; Isely 1944). Ethanol preserved specimens of adult *Sigaus* grasshoppers were used for the mandible analysis. The incisor length (PP‐ID), hinge length (PP‐AP), and molar length (AP‐MD) of the on the posterior surface (Figure 3a) of the left mandible (Patterson 1983, 1984), and body length of each specimen was measured using a Leica stereo microscope (auM225, Olympus, Japan) equipped with a digital camera (SC180, Olympus, Japan) and imaging software (NIS‐Elements 5.01, Nikon Instruments Inc., USA). For consistency, measurement points were aligned under the microscope in the same plane perpendicular to the optical axis of the microscope lens by adjusting the position to bring all points into the same focal plane. Each measurement was taken two or three times on separate occasions, and the average was used as the final measurement for analysis.

The extent of sclerotisation of mandibles (Figure 3b **–**d) was quantified by photographing the anterior surface of the left mandible after mounting on a glass slide. Images of the mandibles were acquired with a Leica stereo microscope (auM225, Olympus, Japan) equipped with a digital camera (SC180, Olympus, Japan) using the same illumination conditions for all specimens. ImageJ (LOCI, University of Wisconsin in Fiji) was used to threshold and analyse the images using the approach of Siegenthaler et al. (2017) with some modifications of the functions used (details in Figure S1). The brightness threshold was set to 0 as minimum and 100 as maximum (0 = pure black, 255 = pure white) using the extent of darkening as a proxy for sclerotisation that involves impregnation with melanin and heavy metals (Krenn 2019).

Statistical analyses and graphing were performed in the R statistics environment (R Core Team 2023) via R Studio 4.0.3 (Boston, MA, USA). Statistical normality was tested using the Shapiro–Wilk test for incisor length, body length, and melanised area. Differences in these three variables among species and sexes were estimated by a linear model, with a posthoc Tukey honest significant difference test (Tukey HSD) for multiple comparisons.

### 2.3 Diet Microhistology

Grasshoppers for diet analysis using microhistology of gut contents were collected in 17 vegetation plots (10 × 20 m) in the Craigieburn Range in early December 1968, late January 1969, March 1969 and January 1970 to understand available plants in grasshopper habitats. The same plots were used for vegetation analysis (see Section 2.4). This provided a total of 1341 grasshoppers (213 female and 177 male *S. nivalis* ; 315 female and 266 male *S. nitidus* ; and 234 female and 136 male *S. australis* ; Table S2).

A reference collection of plant species growing in the vegetation plots was prepared from tissue representing upper and lower leaf surfaces, leaf margin, costal, intercostal and midrib areas that were cleared, stained with 10% carbol fuchsin and mounted on microscope slides (Figure S2; Purchas 1975). The number of grasshoppers sampled per plot ranged from 30 to 154. Gut contents of each were prepared on microscope slides without staining and scanned systematically. Using the reference collection of plant tissues, the plant source of particles in the gut contents were identified and a list of ingested plant species recorded for each grasshopper.

The Ingestion Frequency (IF) of each plant species was calculated as the number of grasshoppers containing that plant species as a proportion of the grasshoppers examined per plot. The IF of flowers and fruits were also recorded between December and March 1969. To compare the diet of grasshopper species in our sample, we combined data of the same sex and species within the same vegetation plot. To reduce the stress value for further nonmetric multidimensional scaling (NMDS) analysis (see Section 2.5), we aggregated the data to plant genera identified in grasshopper gut contents (*n* = 66). The number of vegetation plots contributing data for each of the grasshopper species and sex deviated from the maximum (17) and was adjusted accordingly for NMDS: *S. australis* (15 male, 14 female), *S. nitidus* (16 male, 16 female), *S. nivalis* (11 male, 11 female).

### 2.4 Vegetation Analysis

Plant composition was documented at 17 vegetation plots (10 × 20 m) in the Craigieburn Range that encompassed variation in slope, aspect and ground cover. After collection of grasshoppers, vegetation data (number of plant species and quantitative estimate of species composition) were obtained using the point‐intercept method from 25 randomly allocated sample points per plot (Scott 1965). Vegetation Frequency (VF) for each plant species was estimated as the number of sampling points (out of 25) at which that species was recorded. Dietary preference of grasshoppers was estimated as the ratio between Ingestion Frequency in grasshoppers from plots and Vegetation Frequency in plots (IF/VF): where grasshoppers fed in response to plant availability (IF = IV), avoided a particular plant species (IF < VF) or preferred a particular plant species (IF > VF).

We pooled data for three *Sigaus* species to compare plant richness of their diet with local plant species richness. The number of plant species found per plot (plant richness) and the number of plants eaten by grasshoppers were subjected to regression analysis to test for significant correlations.

### 2.5 Diet DNA Metabarcoding

For DNA metabarcoding, 28 samples (5 female and 4 male *S. nivalis* ; 6 female and 4 male *S. nitidus* ; and 6 female and 3 male *S. australis* ) collected in February and March 2021–2023 from Mount Hutt, Foggy Peak and Craigieburn Range (Table S2). At each site, adult grasshoppers of all three species were caught within an area of < 100 m^2^. This sampling of all three species at three alpine locations allowed us to determine whether sex, species or location had the greater influence on diet composition (Figure 2). Any differences detected among species and sexes would not be due to their access to plants but due to selective feeding, while location differences would be due to the availability of local plants. Sample sizes for metabarcoding were smaller than for microhistology because metabarcoding is more expensive and more sensitive, revealing a larger number of plant species per sample than is possible using cuticle identification.

Specimens were preserved in 99% ethanol with an incised abdomen to maximise preservation of DNA in the gut. Gut contents of 28 grasshoppers were dissected out, and DNA was isolated using the GeneJET Plant DNA extraction Kit (Thermo Fisher Scientific) according to the manufacturer's instructions. Initially, polymerase chain reaction (PCR) amplified two segments of the chloroplast genome, *trn*L intron and *rbc*L (Erickson et al. 2017; Kress and Erickson 2007; Table S4) but taxonomic resolution was best for *trn*L (Figure S4; Tables S5 and S6). Data for analysis were therefore generated using the PCR primers *trn*L‐c A49325 (forward: GAAATCGGTAGACGCTACG) and *trn*L‐d B49863 (reverse: GGGGATAGAGGGACTTGAAC) (Taberlet et al. 2007), yielding fragments of approximately 500 bp.

Amplicons were sequenced on a NovaSeq platform providing 250 bp paired end (PE) reads for > 50,000 tags per sample. Resulting sequences were filtered and denoised to produce amplicon sequence variants (ASVs) using *DADA2* (Callahan et al. 2016) in the QIIME2 (version 2024.5) platform (Bolyen et al. 2019) (Table S4). Limited overlap of forward and reverse PE sequence reads resulted in a low merging rate (< 10% for most of the samples); so we analysed just the forward reads. An optimal truncation of the 5′ end was set at 210 bp (p‐trunc‐len 210), following exploration (e.g., p‐trunc‐len 0 or p‐trunc‐len 200). Identification of plant taxa used comparison of ASVs with a global custom QIIME2 *trn*L reference database created using the DB4Q2 workflow (Dubois et al. 2022) with FASTA nucleotide data obtained from the NCBI database (https://www.ncbi.nlm.nih.gov/) accessed on September 2024 using the *Entrez* text query:(viridiplantae[Organism] AND (trnL[Gene Name] OR tRNA-Leu[Title] OR (trnL-trnF[Title] AND intergenic spacer[Title]) OR trnL[Title] AND 100:500000[Sequence Length]) AND (chloroplast[Gene Name]) OR chloroplast[Title])


Additional *trn*L DNA reference sequences were generated using PCR with the same primers and Sanger sequenced with *trn*L‐c A49325 for vouchers of four plant species (*Anisotome aromatica*, *Aciphylla monroi*, *Celmisia lyallii*, *Celmisia spectabilis* ) from Craigieburn Range and added to our custom reference database and to the NCBI GenBank database (Accessions: PV101254, PV101255, PV101256, PV101257).

The *trn*L ASVs were taxonomically assigned using BLAST+ (classify‐consensus‐blast) with the custom reference database. ASV sequences that were not identified using the QIIME2 BLAST+ algorithm were further examined with the megablast function on the NCBI website to obtain closest matches. Species level identification was assigned only when a 100% pairwise sequence identity was found for species present in New Zealand. The presence of plant taxa identified in this way in the Southern Alps was confirmed using New Zealand Plant Conservation Network database (https://www.nzpcn.org.nz/) and iNaturalist (https://www.inaturalist.org/). The proportion of plant sequences from grasshopper guts assigned to particular plant family, genus and species was recorded.

### 2.6 Diet Differentiation

Inferred plant use by grasshoppers was compared among sampling locations (Mount Hutt, Craigieburn Range, Foggy Peak), species and sexes using a Permutational multivariate analysis of variance (PERMANOVA) (Li et al. 2024; Vicente‐gonzalez et al. 2022) and a nonmetric multidimensional scaling (NMDS) with the R package ‘vegan’ (Oksanen et al. 2022). ASVs comprising less than 1% of reads in 28 samples were removed from the analysis to focus on the major components of grasshopper diet and reduce the stress value in NMDS analysis. Elements of the diet that distinguish sample location, species or sex were identified using PERMANOVA, followed by Kruskal‐Wallis (for locations and species) or Mann–Whitney U Test (for sexes) tests for significant differences in the proportions of plant ASVs (metabarcoding), or plant Ingestion Frequency (microhistology).

## 3 Results

### 3.1 Mandible Morphology

**FIGURE 4:**
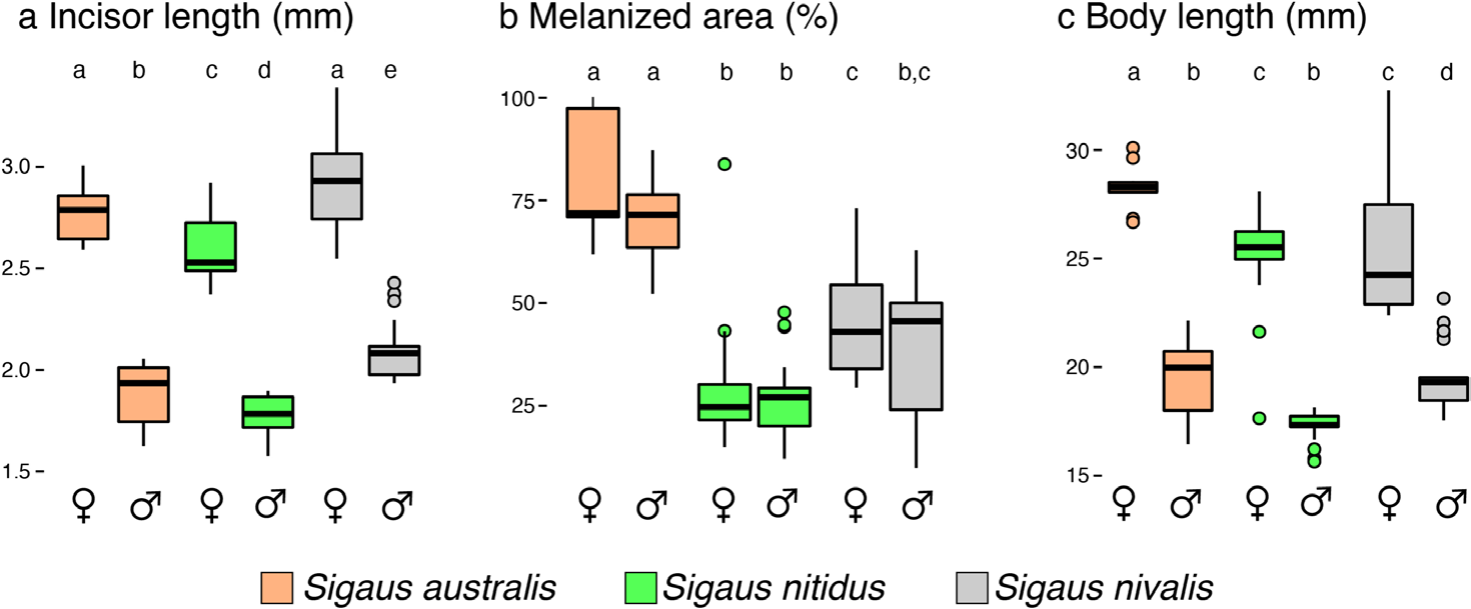
Variation in mandible traits in three sympatric New Zealand alpine grasshoppers among species and sex. Vertical bars indicate standard error. Letters above distribution plots indicate significant differences among all sex × species groups. Differences across groups were assessed using a linear model followed by pairwise comparisons with a Tukey honest significant difference test.

Morphometric data were obtained from mandibles of 124 adult grasshoppers from three *Sigaus* species collected at three locations where they co‐occur. Linear models showed significant differences in body length, incisor, molar and articulation hinge lengths, and melanised area (all *p* < 0.001) among species and sex. In pairwise comparisons using Tukey's HSD, females of all three species had significantly longer incisors ( *S. nivalis* : 2.8 ± 0.3 mm in females, 2.0 ± 0.2 mm in males, *p* < 0.001; *S. nitidus* : 2.4 ± 0.1 mm in females, 1.7 ± 0.1 mm in males, *p* < 0.001; *S. australis*: 2.6 ± 0.3 in females, 1.8 ± 0.2 in males, *p* < 0.001) and larger bodies ( *S. nivalis* : 24.7 ± 23.3 mm in females, 15.2 ± 12.6 mm in males, *p* = 0.01; *S. nitidus* : 28.1 ± 14.9 mm in females, 17.6 ± 4.1 mm in males, *p* < 0.001; *S. australis* : 35.3 ± 5.6 mm in females, 13.8 ± 8.5 mm in males, *p* = 0.002) than males (Figure 4a,c). Articulation hinge and molar lengths were also significantly longer in females compared to males in all three species (Figure S3). No significant differences were observed between the sexes in melanised area ( *S. nivalis* : 48.3% ± 12.4% in females, 42.6% ± 12.6% in males, *p* = 0.8; *S. nitidus* : 38.4% ± 18.5% in females, 44.7% ± 18.9% in males, *p* = 0.7; *S. australis* : 79.0% ± 13.4% in females, 74.8% ± 9.3% in males, *p* = 0.9; Figure 4b). The mandibles of *S. australis* had a significantly larger melanised area than the other two grasshopper species (*p* < 0.001; Figure 4b). These divergences among species and between sexes in mandible traits and body size were confirmed by Principal Component Analysis (Figure S3).

### 3.2 Diet

#### 3.2.1 Microhistology of Gut Contents

**FIGURE 5:**
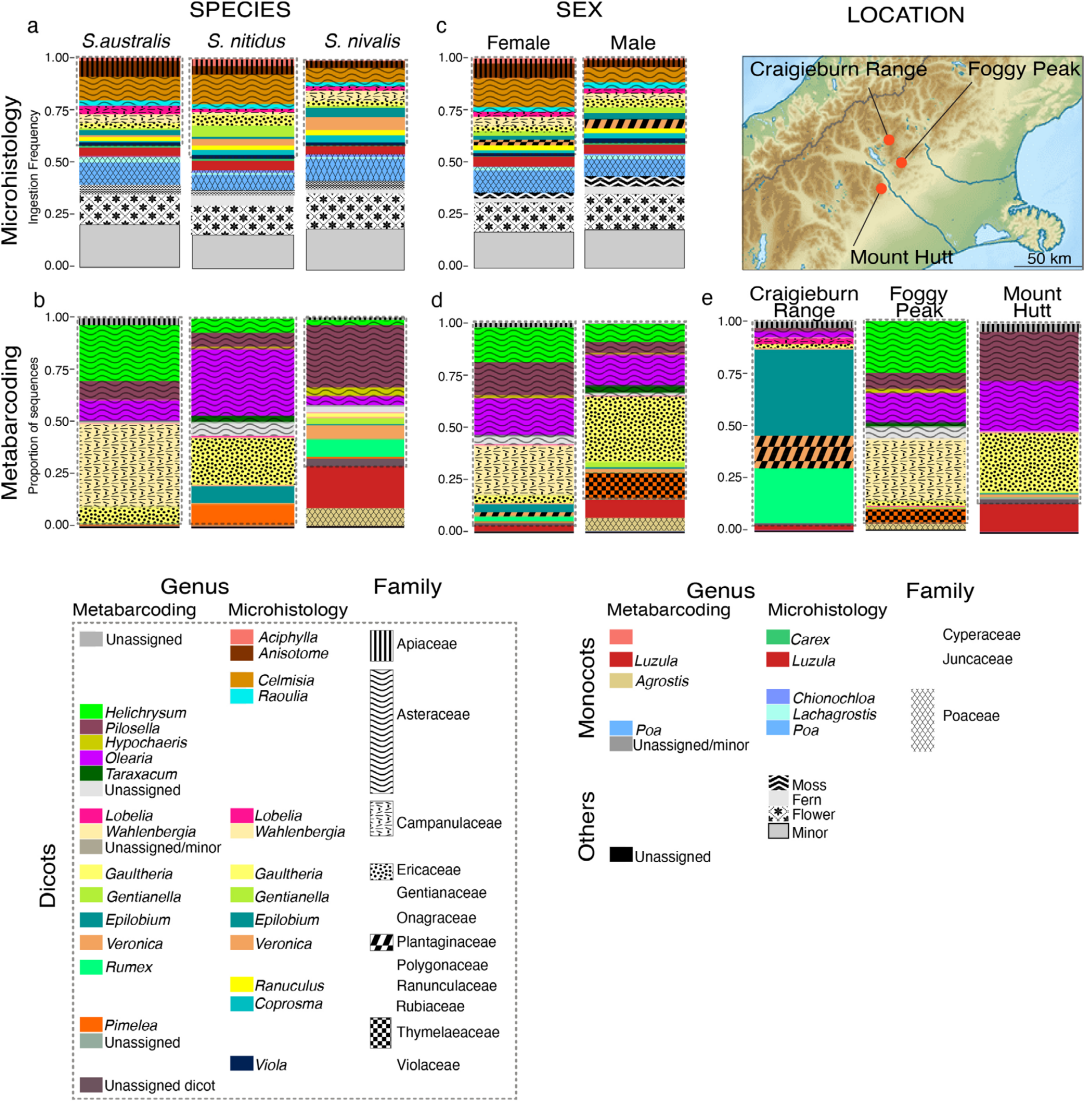
Plant taxa identified from gut contents of New Zealand alpine *Sigaus* grasshopper species using microhistology (1968–1970 samples) DNA metabarcoding (2021–2023 samples). (a, b) Among species; (c, d) between sexes; and (e) among three locations. Different plant genera are represented by colours and plant families by an overlaid pattern.

More than 100 different plant species were identified through microhistological analysis of gut contents from 1341 grasshoppers collected in the Craigieburn Range (Figure 5a,c; Table S3). Most (90%) of these grasshoppers had between one and four plant species in their gut contents, with a maximum of eight detected in one individual. Overall, approximately 43% of plant fragments were from dicotyledonous plants, 17% monocotyledonous plants, 15% were flower parts (of which 28% were identified as *Anisotome aromatica*, the rest unknown), 7% from ferns and mosses, and < 1% from *Gaultheria depressa* fruits. The most commonly eaten plants in the 1968–1970 sample were native species including herbs (*Anisotome aromatica*, *Celmisia* spp.), shrubs (*Coprosma* spp.) and grasses (*Luzula rufa*, *Poa colensoi*) (Figure 5a,c). Samples from different months showed that ingestion frequency of flowers increased through the flowering season. In early summer (December), fewer than 1% of grasshoppers ate flowers, but in January more than half (55%) had eaten, and 6% in March (see Figure S5). Similarly, ingestion frequency of fruits was low in early summer (December and January) and highest (10%) in late summer (March) (Figure S5).

**FIGURE 6:**
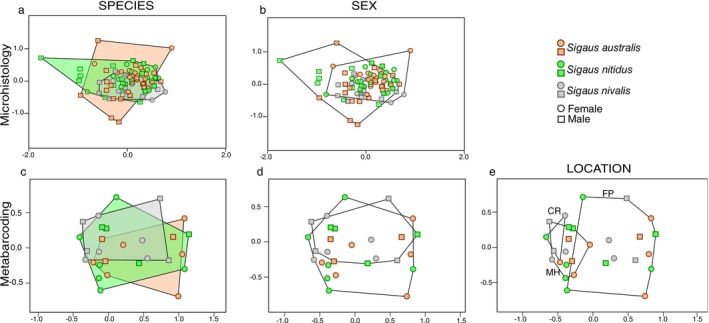
Dietary variation in three New Zealand alpine *Sigaus* grasshopper species on three mountains. Nonmetric multidimensional scaling (NMDS) analysis of microhistological ingestion frequency of 66 plant genera (1968–1970) and metabarcoding with *trn*L relative read abundance of major diet components (28 plant amplicon sequence variants; 2021–2023). Gut content composition among (a, b) species, (c, d) sex, and (e) three populations, Mount Hutt (MH), Craigieburn Range (CR), and Foggy Peak (FP). Polygons encompass all points corresponding to all specimens of each species.

Significant difference was observed in plants eaten among species (PERMANOVA, *p* = 0.001, *R* ^2^ = 0.06, *F* = 2.84, df = 2) and between sexes (*p* = 0.001, *R* ^2^ = 0.04, *F* = 3.54, df = 1) from these microhistology data, but nonmetric multidimensional scaling (NMDS) showed substantial overlap (Figure 6a,b). *S. nivalis* ate fewer plant genera than either *S. australis* or *S. nitidus* (Figure 6a). Overall, male grasshoppers in the sample ate Mountain daisy (*Celmisia* species) slightly less often than female grasshoppers (about 7% less; *p* < 0.05, Mann–Whitney U Test; Figure 5c), but no plant species were eaten exclusively by one grasshopper species or sex.

#### 3.2.2 DNA Metabarcoding of Gut Contents

From DNA in gut contents of 28 grasshoppers, we obtained 7,575,144 sequences (after filtering, denoising and chimera removal), of which an average of 82.5% (±26% SD), could be assigned to plants. Among these, 328 plant (Viridiplantae: Streptophyta) amplicon sequence variants (ASVs) were identified. The *trn*L gene is relatively conserved and so short sequences (210 bp) are sometimes shared among sister species (e.g., within *Chionochloa, Gaultheria, Podocarpus*: see DRYAD repository) and even among genera (e.g., between *Lolium* and *Festuca*). As a result, even 100% identify between ASVs and reference data can yield ambiguous assignments. Eleven ASVs could, however, be confidently assigned at species level: *Agrostis capillaris* (Poaceae), *Hypochaeris radicata* , *Taraxacum officinale* (Asteraceae), *Coriaria arborea* (Coriaraceae), *Calluna vulgaris* (Ericaceae), *Rumex acetosella* (Polygonaceae), *Epilobium glabellum*, *Epilobium melanocaulon* (Onagraceae), *Wahlenbergia albomarginata* (Campanulaceae), *Polytrichastrum alpinum* , (Polytrichaceae), *Brachythecium salebrosum* (Brachytheciaceae). Of the 328 individual ASVs, 202 were assigned to 38 plant genera and the remainder of the ASVs were assigned only to family level (22 families represented). Metabarcoding data revealed between three and 12 genera per grasshopper gut sample. More than 90% of ASVs from *S. australis* and *S. nitidus* were dicotyledons, while *S. nivalis* had consumed a smaller proportion (75%) of dicotyledons and more monocotyledons (25%) including *Agrostis* grass and *Luzula* rush (Figure 5b). The plant taxa most commonly represented in grasshopper gut contents were *Gaultheria* (Ericaceae), *Olearia*, *Raoulia*, and *Pilosella*/*Hieracium* (Asteraceae), *Wahlenbergia* (Campanulaceae), *Luzula* (Juncaceae) and *Agrostis* (Poaceae), comprising > 50% of total sequences in some individuals (Figure 5b,d,e). Invasive herbs from six genera were detected in the grasshopper diet including *Pilosella* and *Hieracium* which could not be differentiated, as some ASV sequences showed 100% pairwise identity with both genera (formerly subgenera).

Nonmetric multidimensional scaling (NMDS) analysis applied to the relative read abundance of 28 ASVs that contributed > 1% of reads in the dataset revealed extensive overlap of diet among grasshopper species and sex (Figure 6c,d). No ASVs were unique to a species or sex, and no significant differences were observed among species (PERMANOVA, *p* = 0.39, df = 2, *R* ^2^ = 0.08, *F* = 1.04) or between sexes (*p* = 0.91, df = 1, *R* ^2^ = 0.02, *F* = 0.60). Greater difference was found among location samples (PERMANOVA, *p* < 0.001, df = 2, *R* ^2^ = 0.18, *F* = 2.68; Figure 6e), with *Gaultheria*, *Olearia*, *Pilosella/Hieracium*, and *Luzula* significantly more abundant in Mt. Hutt and Foggy Peak samples, while *Helichrysum* was found only in Foggy Peak samples (Kruskal‐Wallis test, all *p* < 0.01; Figure 5e).

### 3.3 Plant Selection by the Grasshopper Guild

**FIGURE 7:**
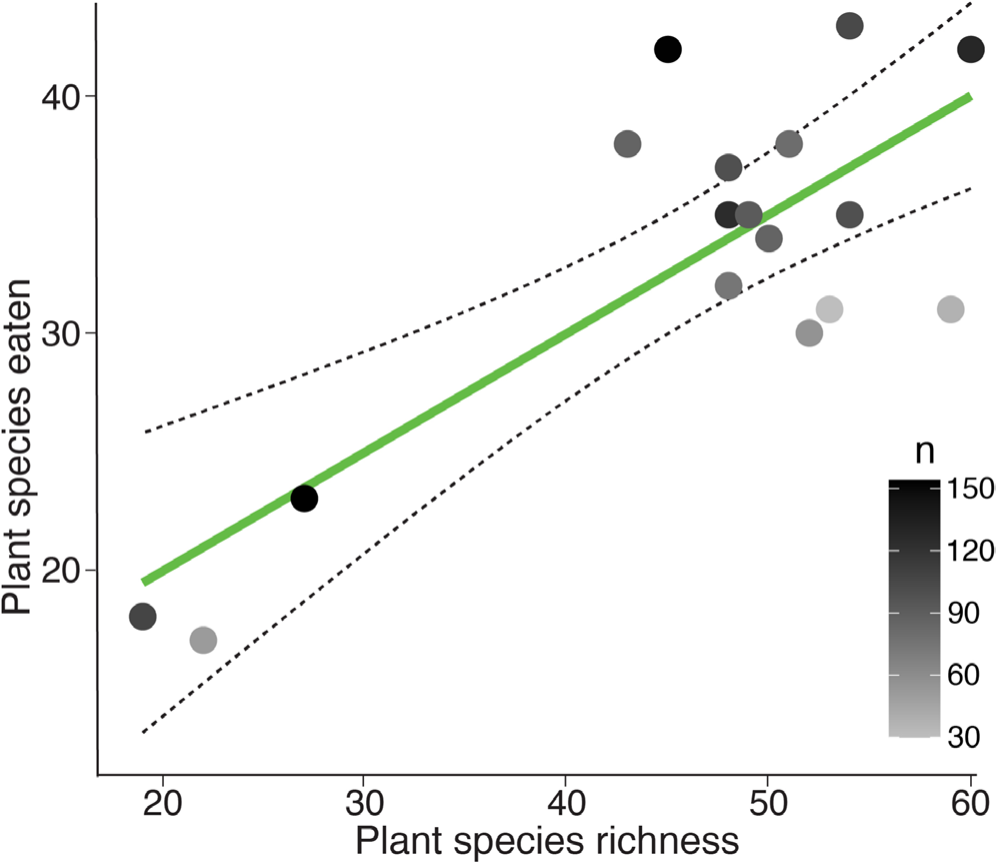
Positive relationship (*R* ^2^ = 0.62, *F* = 24.72, *p* < 0.0001) between plant richness and the number of plant species identified using microhistology from gut contents of *Sigaus* spp. alpine grasshoppers sampled in 17 vegetation plots (10 X 20m) in the Craigieburn Range, New Zealand. Grey gradient fill indicates the number of grasshoppers sampled for each plot.

Given the limited differentiation of diet among the three sympatric grasshopper species and the wide range of plant taxa they consumed, we considered them as a guild and investigated whether there was any evidence of selective feeding. Using the ingestion frequency (IF) from microhistology data for 1341 grasshoppers collected from vegetation plots in the Craigieburn Range, and analysis of vegetation frequency (VF) in the same plots, we found a significant positive relationship between the number of plant species available in a vegetation plot and the number eaten (number of plant species eaten = 9.88 [−0.34, 20.1] + 0.50 [0.29, 0.72] × Plant species richness; *R* ^2^ = 0.62, *F*‐statistic = 24.72, *p* < 0.0001; Figure 7).

**FIGURE 8:**
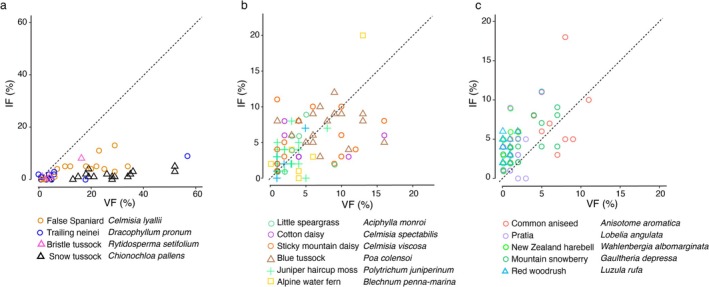
Food preferences of the alpine *Sigaus* grasshopper guild. Comparison of (a) low, (b) medium, and (c) high plant ingestion frequency (IF) by grasshoppers with vegetation frequency (VF) among vegetation plots in the Craigieburn Range, New Zealand in 1968–1970. Each point represents a plant species from a vegetation plot, with the grey line indicating an equal relationship between ingestion frequency and vegetation frequency.

The ingestion frequency (IF) of plant species was compared to the vegetation frequency (VF) to assess whether the grasshoppers consumed plants in proportion to their availability or were more selective about plants eaten (Figure 8). We found evidence that some plant species were avoided. The alpine zone snow tussock ( *Chionochloa pallens* ) had on average 30% vegetation frequency in 17 plots, but was rare (1.2%) in grasshopper gut contents (IF<VF) (Figure 8a). This and other plants (*Celmisia lyallii*, *Dracophyllum pronum*, *Rytidosperma setifolium* ) with IF < VF (Figure 8a) have particularly tough tissues . Plant species that were more common than expected in grasshopper guts (IF > VF) were soft‐leaved ground‐cover species (*Anisotome aromatica, Lobelia angulata, Gaultheria depressa, Wahlenbergia albomarginata, Luzula rufa*). These plant species occupied a small proportion of vegetation plots (< 5% and many < 1% VF) but were common food species (high IF) in more than 75% of plots (Figure 8c). Many other plant species were eaten in similar proportions to their availability in the sampled environment (IF = VF) including herbs (*Aciphylla monroi, Celmisia spectabilis*, *Celmisia viscosa*), native blue grass (*Poa colensoi*), a moss ( *Polytrichum juniperinum* ) and a fern (*Blechnum penna‐marina*) (Figure 8b).

### 3.4 Longitudinal Comparison of Diet

The microhistological data were collected 50 years earlier than the samples used for metabarcoding. Despite differences in time and location of sampling, all analyses showed that the endemic snowberries (*Gaultheria*), harebells (*Wahlenbergia*), pratia (*Lobelia*), and woodrush (*Luzula*) were prominent components of grasshopper diet (Table 1, Figures 5 and 8c), while hard‐tissue plants such as false Spaniard (*Celmisia lyallii*) and snow tussock ( *Chionochloa pallens* ) were minor food sources (Figure 8a) despite their high abundance in the alpine habitat.

**TABLE 1:** Plant families and genera recorded from gut content analysis of New Zealand alpine *Sigaus* grasshoppers using microhistological analysis (1968–1970) and DNA metabarcoding with *trn*L (2021–2023). Only major components of the grasshopper diet are shown (full list in Supplementary/Dryad). I = introduced invasive plant species in New Zealand. M = plants that are major components of diet (Figure 6), or trace. H = herbs, S = shrubs, G = grasses.

	Family	Genus	Common name	Growth form	1968–70 histology	2021–23 trnL
Dicotyledons	Apiaceae	*Aciphylla*	Speargrass	H	M	Trace
		*Anisotome*		H	M	
		Unidentified		H		M
	Asteraceae	*Celmisia*	Alpine daisy	H, S	M	Trace
		*Cirsium*	Thistle	H		Trace
		*Raoulia*	Cushion	H	M	M
		*Pilosella/Hieracium*	Hawkweed	H		M
		*Hypochaeris*	Cat's ear	H		M
		*Olearia*	Daisy‐bush	H		M
		*Taraxacum*	Dandelion	H		M
	Campanulaceae	*Lobelia*	Pratia	H	M	M
		*Wahlenbergia*	Harebell	H	M	M
	Coriariaceae	*Coriaria*	Tutu shrub	S		Trace
	Ericaceae	*Calluna*	Heather	S		Trace
		*Gaultheria*	Snowberry	H, S	M	M
	Fabaceae	*Lupinus*	Lupin	H		Trace
	Gentianaceae	*Gentianella*	Gentian	H	M	M
	Onagraceae	*Epilobium*	Willowherb	H	M	M
	Plantaginaceae	*Veronica*	Hebe	H, S	M	M
	Podocarpaceae	*Podocarpus*	Totara	S		Trace
	Polygonaceae	*Rumex*	Sorrel	H	Trace	M
	Ranunculaceae	*Ranunculus*	Buttercup	H	M	Trace
	Rosaceae	Unidentified		H		Trace
	Rubiaceae	*Coprosma*		H, S	M	
	Thymelaeaceae	*Pimelea*		H, S	Trace	M
	Violaceae	*Viola*		H	M	Trace
Monocotyledons	Poaceae	*Agrostis*	Bent	G	Trace	M
		*Chionochloa*	Tussock grass	G	M	Trace
		*Festuca*	Fescue	G	Trace	
		*Lachnagrostis*	wind grass	G	M	
		*Poa*	Blue grass	G	M	M
	Juncaceae	*Luzula*	Woodrush	G	M	M
	Cyperaceae	*Carex*	Sedge	G	M	
Bryophyta	Andreaeaceae	*Andreaea*	Rock moss			Trace
	Polytrichaceae	*Polytrichum*	Hair moss		M	Trace
Marchantiophyta	Lophocoleaceae	*Lophocolea*	Liverwort			Trace
Pteridophyta			Ferns		M	

Nine plant genera were detected only in the 2021–2023 gut contents, and seven of these were invasive species (Table 1), including the European hawkweeds (*Pilosella*/*Hieracium* spp.), common dandelion ( *Taraxacum officinale* ), and false dandelion ( *Hypochaeris radicata* ) (Figure 5b,d,e). These invasive plant genera were detected in 26 out of 28 gut samples and comprised > 40% of sequences in six samples. Our metabarcoding data from Craigieburn Range had a high proportion of the invasive sheep's sorrel species ( *Rumex acetosella* ) which represented < 1% of plant fragments 50 years earlier. In contrast, endemic *Celmisia, Ranunculus, Viola, Coprosma*, mosses and ferns were identified as major components in 1968–1970 samples (Figure 4a,c; Table 1), but they were minor components or undetected in 2021–2023 samples.

## 4 Discussion

Sympatry of herbivores is sometimes associated with selective feeding that reduces competition for resources (Li et al. 2024). Three species of *Sigaus* grasshoppers are abundant in the Southern Alps alpine zone, a habitat of snow tussock grasses, small shrubs, herbs and cushion plants (Chinn and Chinn 2020; Jia et al. 2018) and thus provide insight into insect herbivory in this endemic system. The mandibles of the three grasshopper species showed variation in sclerotisation and females were significantly larger than males, suggesting potential adaptation to food plants of different sizes and toughness. However, we found greater diet differences among locations than among grasshopper species. All three *Sigaus* grasshoppers consume a wide range of plants with only minor variation among species and sexes, so we combined them into a guild to examine how selective they are on a microscale.

The mandibles of insect herbivores are adapted to their food plants (Clissold 2007; Krenn 2019), and *Sigaus* have mandibles similar to other short‐horn grasshoppers that eat broadleaf plants (Figure 3; Isely 1944; Chapman 1964; Gangwere 1965; Kang et al. 1999). We found that the mandibles of *S. australis* , *S. nitidus* and *S. nivalis* differed more between sexes than among species (Figure 4a). The larger mandibles of females suggest that sexual differences in diet may be more pronounced than species differences, with females able to feed on more robust leaves than males as in other insects such as the stick insect *Megacrania batesii* (Miller et al. 2024) and the grasshopper *Romalea microptera* (Vincent 2006). We found that although male and female *Sigaus* grasshoppers fed on the same range of plants, females ate significantly more of the thick, tomentose leaves of mountain daisy (*Celmisia*) than males.

The extent of sclerotisation of the mandibles did not differ between sexes but was more extensive in *S. australis* than in other *Sigaus* (Figures 3 and 4b). As sclerotisation in insects is related to hardening of the exoskeleton (Krenn 2019), it is possible that *S. australis* can feed on tougher plant tissues and/or maintain functioning mandibles longer than the congenerics. Plant toughness is a physical barrier against herbivores and is well‐demonstrated by experimental addition of silicon to food of the grasshopper *Oxya grandis* to significantly reduce herbivory (Mir et al. 2019). Our microhistology and metabarcoding data suggest that it is *S. nivalis*, not *S. australis* , that eats a greater proportion of the tough monocotyledons (grasses and woodrush) and a narrow range of plant taxa. Despite the differences in mandible sclerotisation in *Sigaus*, no species‐specific food plants were detected. This lack of preference is supported by captive choice experiments in which neither sexual nor species differences were detected among *Sigaus* grasshoppers (Nakano et al. 2024). The distinct mandibles of *S. australis* may be more resistant to wear than the mandibles of sympatric grasshopper species with less sclerotisation (Büsse and Gorb 2018). Despite significant differences in size between males and females of all three grasshopper species (Meza‐Joya et al. 2022), we did not detect sex‐specific host plants. However, microhistology did detect greater consumption of mountain daisy (*Celmisia* species) by females compared to male grasshoppers. It has been observed that incisive strength is positively correlated with the toughness of plants eaten by grasshoppers rather than the size of jaw or molar strength (Ibanez, Lavorel, et al. 2013), so size and sclerotisation might have little influence on cutting strength, and thus be unrelated to tissue traits of plants eaten. The present study, however, did detect variation in *Sigaus* diet among locations, with Mount Hutt grasshoppers eating more *Gaultheria*, *Olearia*, *Pilosella*, and *Luzula*, while *Helichrysum* was included in the diet at Foggy Peak (Figure 5e). Location differences are likely to be associated with local variation in plant species availability and abundance as shown by the positive relationship between plant richness in vegetation plots and eaten by grasshoppers sampled from the vegetation plots.

We used *Sigaus* gut contents for metabarcoding and for microhistology to identify food plants eaten. Leaf fragments could be identified to species level using microhistology but the conserved cpDNA *trn*L fragment limited identification to genus or family level in most cases. Microhistology also enables detection of different plant structures such as seeds, flowers and stalks (Carrière 2002; García‐Gutiérrez et al. 2020). This was important for detection of the large floral component of the diet during seasons (and years) when flowers were abundant; however, species identification is usually only achieved from leaf epidermis (Johnson et al. 1983). Metabarcoding, on the other hand, can yield taxonomically informative data from all cellular material. Together, the two tools should provide a more complete list of food plant species and tissues consumed. In total, more than 100 food plant species were identified in the microhistological analysis while plants belonging to 38 genera and 22 families were obtained from the gut contents of *Sigaus*, confirming that all three grasshopper species are polyphagous.

### 4.1 Do Sigaus Show Dietary Preferences?

The three *Sigaus* species fed on a range of shrubs, herbs, grasses and cryptogams, although individual grasshoppers had only a few species of food plants in their guts (max = 12). The range of species eaten is partly determined by availability as shown by both the location effect (Figure 6e) and the positive correlation between plant richness in the local environment and the number of plant species eaten by local grasshoppers (Figure 7). However, *Sigaus* feeding was not dictated solely by availability as we found some plant species were frequently represented in the gut despite having low abundance in the grasshopper habitat (Figure 8). Favoured food plants, including endemic snowberries (*Gaultheria*), harebells (*Wahlenbergia*), pratia (*Lobelia*), and soft woodrush (*Luzula*), typically comprised < 5% of the vegetation within the surveyed plots (Figure 8). The selection of uncommon plant species might be directly linked if the grasshopper feeding is reducing the abundance of their preferred food. In contrast, abundant plant species in the Craigieburn flora such as hard tussock *Chionochloa pallens* (30%–60%) and false Spaniard *Celmisia lyallii* (10%–25%) contributed < 1% of *Sigaus* diet, indicating these plants are avoiding damage caused by grasshopper feeding (also observed in captivity; Nakano et al. 2024).

Our analyses of *Sigaus* diet from data gathered 50 years apart showed that location had a stronger influence on *Sigaus* diet than either sex or species but that the endemic snowberries, harebells, pratia, and woodrush were consistently important components in the diet of these grasshoppers. Significantly, longitudinal differences in diet resulted primarily from eating introduced invasive weeds in 2021–2023 that were not recorded or rare in the vegetation surveys 50 years earlier (Watson 1970; Day and Buckley 2007). Hawkweed (*Pilosella*), sheep's sorrel ( *Rumex acetosella* ), common dandelion ( *Taraxacum officinale* ), and false dandelion ( *Hypochaeris radicata* ) were commonly detected in *Sigaus* grasshopper guts using metabarcoding (Table 1). These invasive plant species were not observed (or not common) in the grasshopper diet in 1968–1970 collected from Craigieburn. Although there are significant differences in sample size and method, the absence of invasive plant species in 1968–1970 was recorded using vegetation plots as well as gut samples. While equivalent records for 50 years ago are not available for Mt. Hutt and Foggy Peak (where grasshoppers were also collected for metabarcoding), data from 125 repeated vegetation transects in high‐country grasslands across this region (Canterbury and Otago) indicate that an increase in exotic plant species is widespread (Day and Buckley 2007). *Hieracium* (including *Pilosella*) were the main contributors to the increase in exotic species richness in tussock grasslands over the 24‐year period. *Hieracium* species are invasive weeds in the New Zealand high country and have increased their distribution and abundance in the past 50 years (Jensen et al. 1997; Meffin 2010; Steer and Norton 2013), and thus only recently have come available to these alpine grasshoppers. The increase in *Hieracium* species in New Zealand tussock grasslands does not appear to be directly related to land use changes, management practices nor native plant species composition (Day and Buckley 2007). However, colonisation of disturbed sites is known to be rapid (French 2021) and an increase in exotic plant species has been documented at all tussock grassland sites that provide habitat for *Sigaus* grasshoppers (Day and Buckley 2007; Rose et al. 2004).

Herbivores have the potential to influence species evenness and richness of plant communities (Jia et al. 2018). Together our data demonstrate that these alpine grasshoppers are broadly polyphagous and responsive to plant species and plant structures available in their environment throughout the year. Nevertheless, *Sigaus* grasshoppers also demonstrate selectivity and clear preference for certain palatable species and rejection of others, as previously indicated (Nakano et al. 2024). In particular, although *Sigaus* are abundant among the *Chionochloa* tussock grasses that dominate (in both number and biomass) in much of the alpine habitat, they rarely eat them (Figure 8). As *Sigaus* grasshoppers represent the majority of invertebrate biomass in the New Zealand alpine environment (Chinn and Chinn 2020; White 1974b, ; White and Watson 1972) and prefer to eat soft herbs, shrubs and their flowers (including introduced weeds), it is likely that they have a significant influence on the plant communities in New Zealand alpine areas.

## Author Contributions

**Mari Nakano:** conceptualization (equal), data curation (lead), formal analysis (lead), funding acquisition (equal), investigation (equal), methodology (equal), project administration (equal), resources (equal), software (lead), supervision (supporting), validation (equal), visualization (equal), writing – original draft (lead), writing – review and editing (equal). **Mary Morgan‐Richards:** conceptualization (equal), data curation (lead), formal analysis (equal), funding acquisition (equal), investigation (equal), methodology (equal), project administration (equal), resources (equal), software (equal), supervision (lead), validation (equal), visualization (equal), writing – original draft (equal), writing – review and editing (equal). **Steven A. Trewick:** conceptualization (equal), data curation (equal), formal analysis (equal), funding acquisition (equal), investigation (equal), methodology (equal), project administration (equal), resources (equal), software (equal), supervision (lead), validation (equal), visualization (lead), writing – original draft (equal), writing – review and editing (equal). **Richard N. Watson:** data curation (equal), formal analysis (equal), investigation (equal), methodology (supporting), resources (equal).

## Funding

This work was supported by the Entomological Society of New Zealand. Miss E. L. Hellaby Indigenous Grassland Research Trust, The Orthopterists' Society.

## Consent

The authors have nothing to report.

## Conflicts of Interest

The authors declare no conflicts of interest.

## Supporting information


**Figure S1:** Protocol for measuring melanised area of New Zealand alpine grasshoppers. (a) shows diffuser wrapped around the specimen to avoid reflection. ImageJ (LOCI, University of Wisconsin in Fiji) was used to threshold and analyse the images as follows: firstly, the image was converted into an 8‐bit grey‐scale (Image>Type > 8‐bit; (b). Then, the area of interest was selected (with polygon selection; (c), using homologous landmarks present in all species and sexes of the grasshoppers. The brightness threshold was set 0 as minimum and 100 as maximum (0 = pure black and 255 = pure white) using the ‘Threshold’ function (Image>Adjust > Threshold; (d). Finally, using the ‘Analyse Particles’ function (Analyse>Analyse Particles), the proportion (%) of area of interest (yellow line in d) that was in the range of the threshold (red colour in d) was calculated. This method was based on Siegenthaler et al. (2017) with some modifications to the functions used.
**Figure S2:** Examples of New Zealand alpine plants and their leaf epidermal morphology: (A, B) *Chionochloa* spp.; (C,D) *Poa colensoi*; (E,F) *Luzula* spp.; (G,H) *Blechnum penna‐marina*; (I–L) *Celmisia spectabilis* ; (M,N) *Gentianella corymbifera*; (O,P) *Podocarpus nivalis* (and *Sigaus nivalis*); (Q,R) *Gaultheria crassa*; (S,T) *Gaultheria depressa*; (U–X) *Wahlenbergia albomarginata* (V,W adaxial; X abaxial surface). Abbreviations: S = stoma(ta), Ec = epidermal cell, T = trichomes.
**Figure S3:** Variation in the mandible traits (left) and principal component analysis generated using mandible traits (right) in the three *Sigaus* species (*S. nivalis, S. nitidus, S. asutralis*). Left: Vertical bars indicate standard error. Letters above distribution plots indicate significant differences among all sex × species groups. Differences across groups were assessed using a linear model followed by pairwise comparisons with a Tukey honest significant difference test. Right:
**Figure S4:** Proportion of *rbc*L (a) and *trn*L (b) sequences assigned to specific genus and family. Different genera represented in different colours and different families represented in different patterns (if multiple genera are included within a family). Plant comprising less than < 1% of sequences in all samples not shown in the figure: *Polytrichum juniperinum* (Polytrichaceae). Abbreviations: nvF = *Sigaus nivalis* female, nvM = *S. nivalis* male, ntF = *Sigaus nitidus* female, ntM = *S. nitidus* male, auF = *S. australis* female, auM = *S. australis* male.
**Figure S5:** Change in average ingestion frequency (%) of flower parts (including unidentified species and *Anisotome aromatica*) and *Gaultheria depressa* fruits across different months during the summer of 1969 in *Sigaus* grasshoppers from 17 plots examined in the Craigieburn Range.
**Table S1:** Collection sites and year and sample size of New Zealand alpine grasshoppers used for mandible analysis.
**Table S2:** Collection sites (population, geolocation, elevation), year, and sex of the three species of New Zealand alpine grasshoppers. and method used for gut content analysis (DNA metabarcoding or Microhistology).
**Table S3:** List of plant species identified from the crop and gut contents of three *Sigaus* grasshopper species using microhistological epidermal analysis. Specimens were collected between 1968 and 1970. The taxonomic names in brackets are previously used names.
**Table S4:** Plant chloroplast rbcL and trnL sequence reads from PCR amplicons of gut contents of 28 New Zealand alpine grasshoppers collected at Mount Hutt, Broken River and Foggy Peak after filtering, denoising, merging (for rbcL) and removing chimeric sequences.
**Table S5:** Number of taxa identified at family, genus and species levels in each gut content of New Zealand alpine grasshoppers using different identification markers (*rbc*L and *trn*L). Taxa of *rbc*L and *trn*L sequences were identified using a custom reference database and BLAST+ in QIIME2.
**Table S6:** Comparison of plant taxonomic identification from *rbc*L and *trn*L chloroplast fragments to family, genus and species level. Shading indicates a higher taxonomic identification from *rbc*L or *trn*L of the same sample.

## Data Availability

The DNA data generated for this study are available on DRYAD https://doi.org/10.5061/dryad.2rbnzs82g. Sanger sequences for four plant species (*Anisotome aromatica*, *Aciphylla monroi*, *Celmisia lyallii*, *Celmisia spectabilis* ) from Craigieburn Range are available via Genbank (accessions: PV101254, PV101255, PV101256, PV101257).
