# Supplementary material for: Dietary Overlap of Sympatric Polyphagous Alpine Grasshoppers Includes Invasive Plant Species
Source: Ecol Evol. 2026 Apr 29;16(5):e73576. doi: 10.1002/ece3.73576 (PMC13125957; doi:10.1002/ece3.73576)
Supplement: Supplementary file 1 — Figure S1: Protocol for measuring melanised area of New Zealand alpine grasshoppers. (a) shows diffuser wrapped around the specimen to avoid reflection. ImageJ (LOCI, University of Wisconsin in Fiji) was used to threshold and analyse the images as follows: firstly, the image was converted into an 8‐bit grey‐scale (Image>Type > 8‐bit; (b). Then, the area of interest was selected (with polygon selection; (c), using homologous landmarks present in all species and sexes of the grasshoppers. The brightness threshold was set 0 as minimum and 100 as maximum (0 = pure black and 255 = pure white) using the ‘Threshold’ function (Image>Adjust > Threshold; (d). Finally, using the ‘Analyse Particles’ function (Analyse>Analyse Particles), the proportion (%) of area of interest (yellow line in d) that was in the range of the threshold (red colour in d) was calculated. This method was based on Siegenthaler et al. (2017) with some modifications to the functions used. Figure S2: Examples of New Zealand alpine plants and their leaf epidermal morphology: (A, B) Chionochloa spp.; (C,D) Poa colensoi; (E,F) Luzula spp.; (G,H) Blechnum penna‐marina; (I–L) Celmisia spectabilis ; (M,N) Gentianella corymbifera; (O,P) Podocarpus nivalis (and Sigaus nivalis); (Q,R) Gaultheria crassa; (S,T) Gaultheria depressa; (U–X) Wahlenbergia albomarginata (V,W adaxial; X abaxial surface). Abbreviations: S = stoma(ta), Ec = epidermal cell, T = trichomes. Figure S3: Variation in the mandible traits (left) and principal component analysis generated using mandible traits (right) in the three Sigaus species (S. nivalis, S. nitidus, S. asutralis). Left: Vertical bars indicate standard error. Letters above distribution plots indicate significant differences among all sex × species groups. Differences across groups were assessed using a linear model followed by pairwise comparisons with a Tukey honest significant difference test. Right: Figure S4: Proportion of rbcL (a) and trnL (b) sequences assigned to specific g [file ECE3-16-e73576-s001.docx]

Supplementary Information for:

Mandible morphology, molecular and microhistological analyses to understand the food plants of three species of New Zealand *Sigaus* Grasshoppers

Mari Nakano, Steven A. Trewick, Richard N. Watson, Mary Morgan-Richards

**Figures**


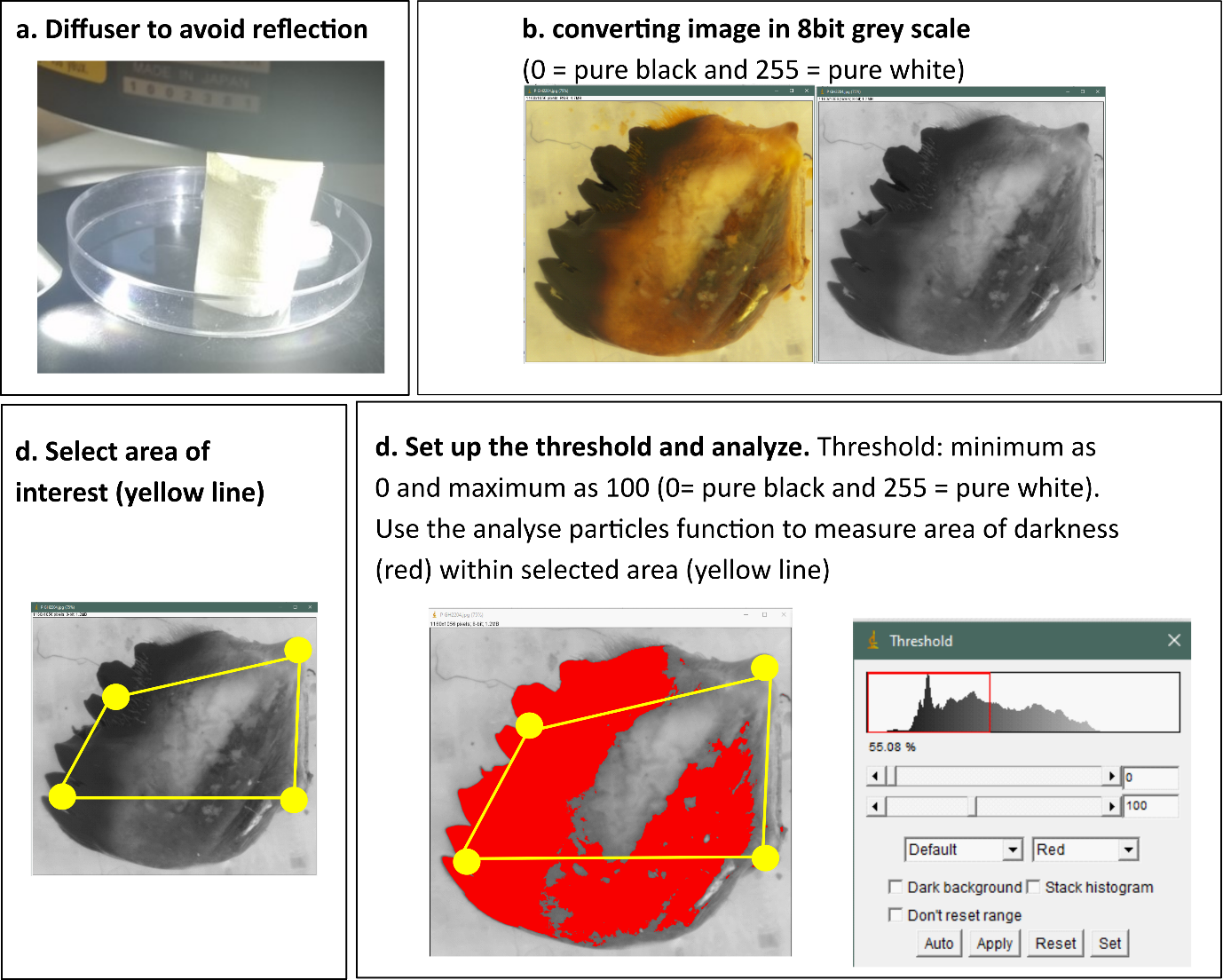


**Figure S1.** Protocol for measuring melanized area of New Zealand alpine grasshoppers. **a** shows diffuser wrapped around the specimen to avoid reflection. ImageJ (LOCI, University of Wisconsin in Fiji) was used to threshold and analyze the images as follows: firstly, the image was converted into an 8-bit grey-scale (Image >Type > 8-bit; **b**). Then, the area of interest was selected (with polygon selection; **c**), using homologous landmarks present in all species and sexes of the grasshoppers. The brightness threshold was set 0 as minimum and 100 as maximum (0 = pure black and 255 = pure white) using the ‘Threshold’ function (Image > Adjust > Threshold; **d**). Finally, using the ‘Analyze Particles’ function (Analyze > Analyze Particles), the proportion (%) of area of interest (yellow line in **d**) that was in the range of the threshold (red colour in **d**) was calculated. This method was based on Siegenthaler et al. (2017) with some modifications to the functions used.


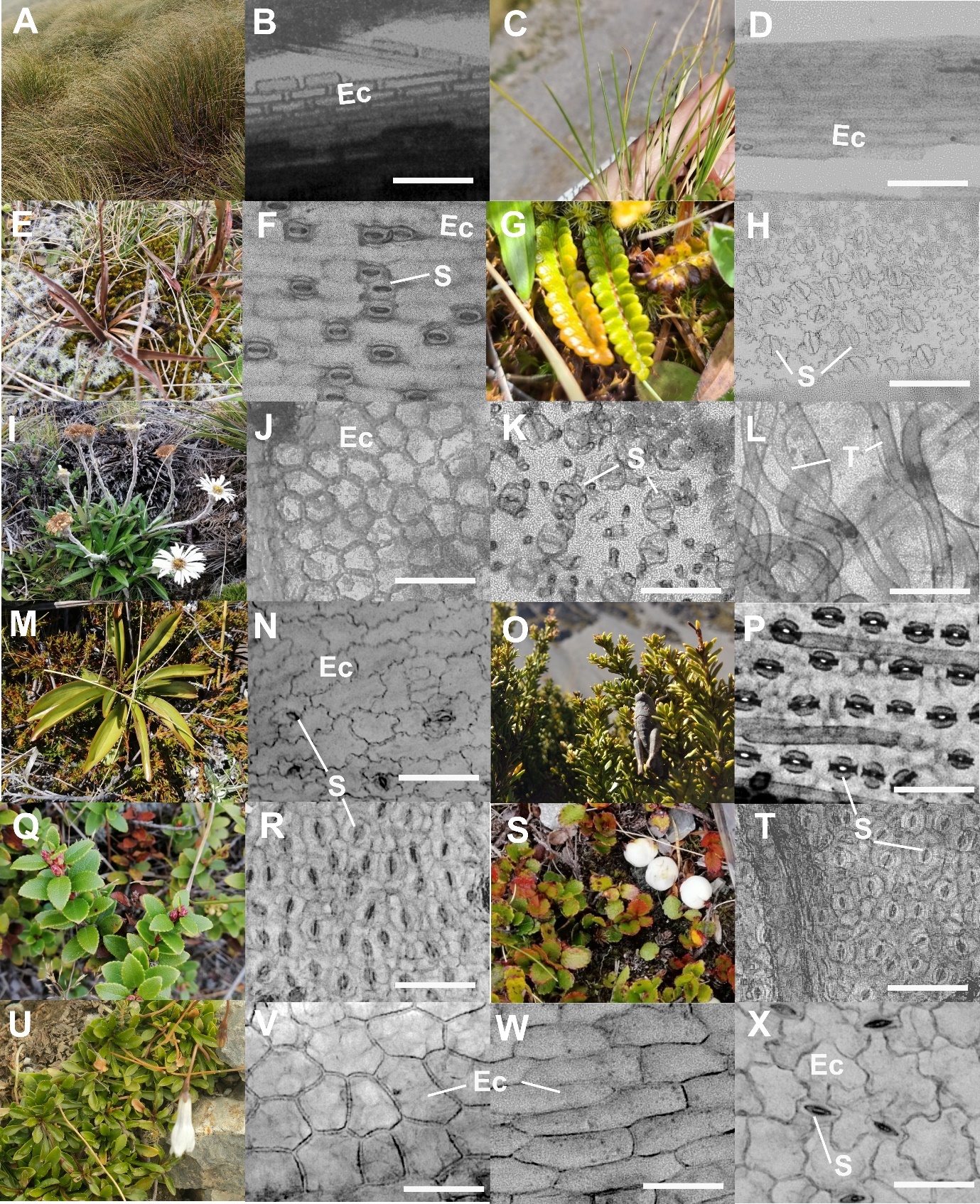


**Figure S2.** Examples of New Zealand alpine plants and their leaf epidermal morphology: **A, B** Chionochloa spp.; **C,D** Poa colensoi; **E,F** Luzula spp.; **G,H** Blechnum penna-marina; **I–L** Celmisia spectabilis; **M,N** Gentianella corymbifera; **O,P** Podocarpus nivalis (and Sigaus nivalis); **Q,R** Gaultheria crassa; **S,T** Gaultheria depressa; **U–X** Wahlenbergia albomarginata (**V,W** adaxial; **X** abaxial surface). Abbreviations: S = stoma(ta), Ec = epidermal cell, T = trichomes.


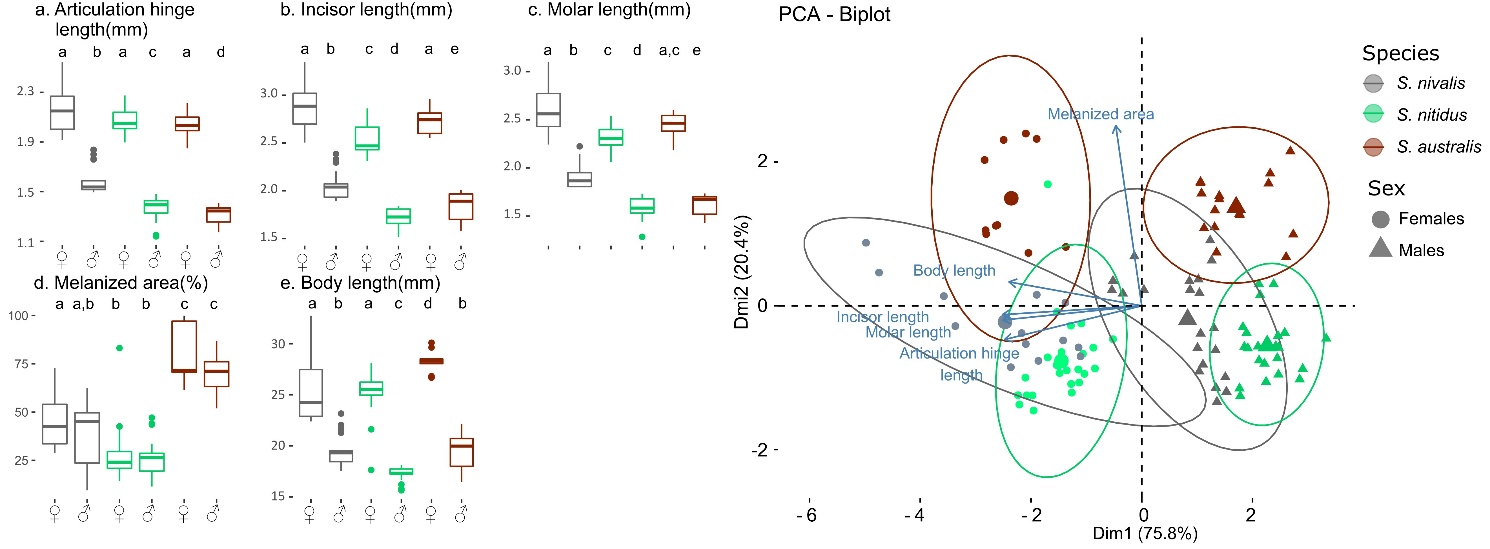


**Figure S3.** Variation in the mandible traits (left) and principal component analysis generated using mandible traits (right) in the three *Sigaus* species (*S. nivalis, S. nitidus, S. asutralis*). Left: Vertical bars indicate standard error. Letters above distribution plots indicate significant differences among all sex x species groups. Differences across groups were assessed using a linear model followed by pairwise comparisons with a Tukey honest significant difference test. Right:


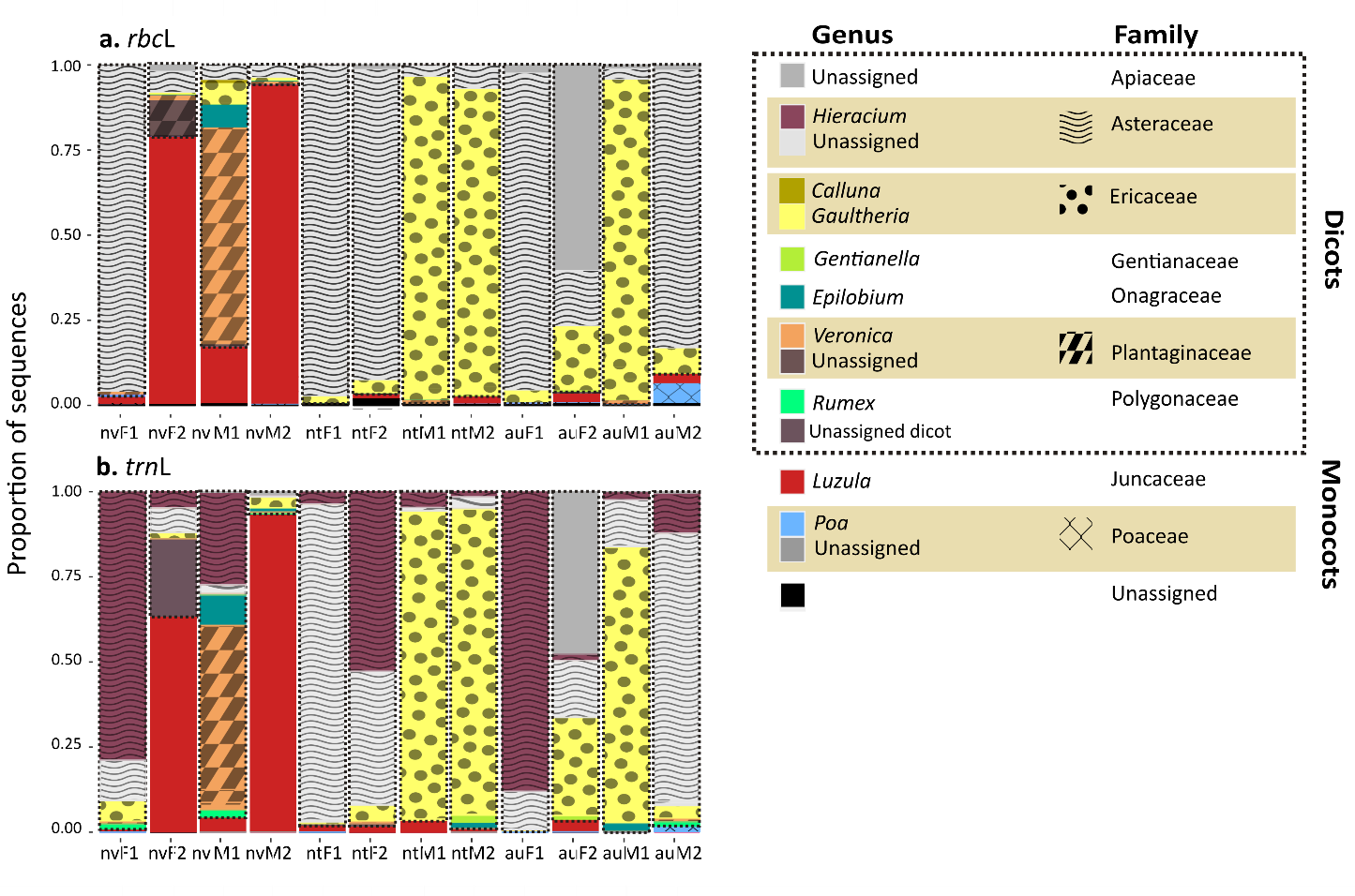


**Figure S4.** Proportion of *rbc*L (**a**) and *trn*L (**b**) sequences assigned to specific genus and family. Different genera represented in different colors and different families represented in different patterns (if multiple genera are included within a family). Plant comprising less than <1% of sequences in all samples not shown in the figure: *Polytrichum juniperinum* (Polytrichaceae). Abbreviations: nvF = *Sigaus nivalis* female, nvM = *S. nivalis* male, ntF = *Sigaus nitidus* female, ntM = *S. nitidus* male, auF = *S. australis* female, auM = *S. australis* male.


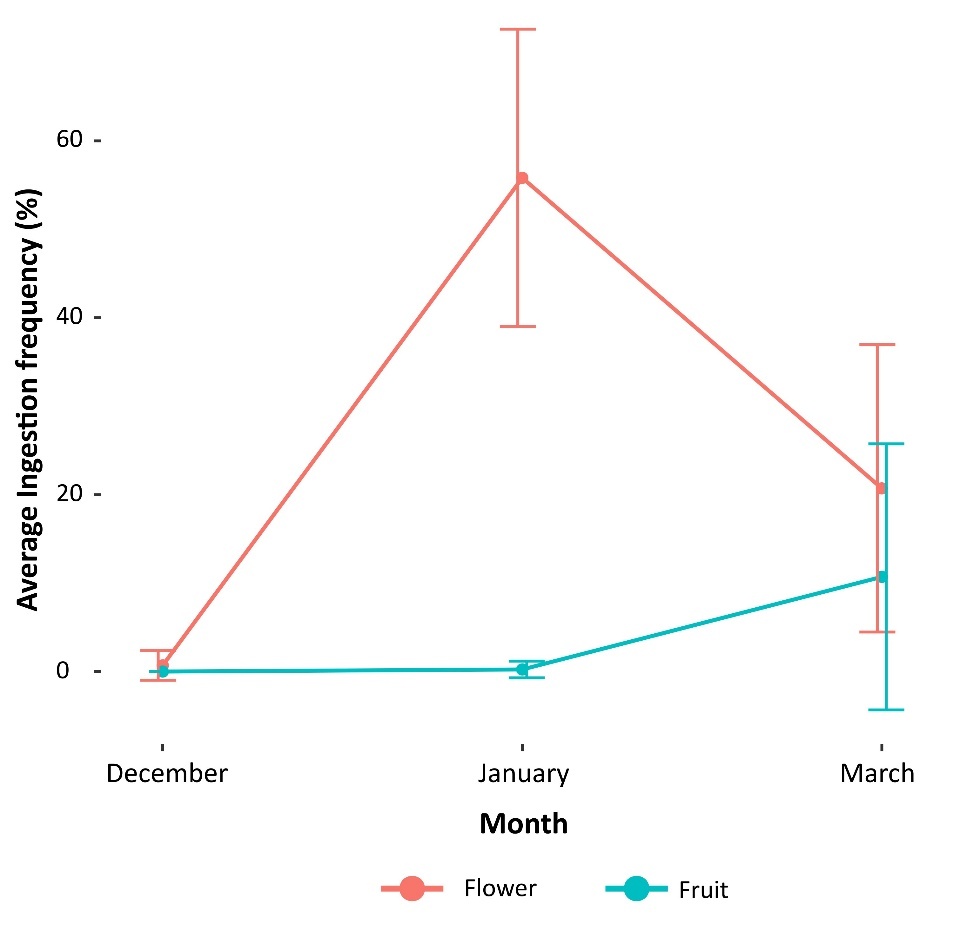


**Figure S5.** Change in average ingestion frequency (%) of flower parts (including unidentified species and Anisotome aromatica) and Gaultheria depressa fruits across different months during the summer of 1969 in Sigaus grasshoppers from 17 plots examined in the Craigieburn Range

**Tables**

**Table S1.** Collected sites and year and sample size of New Zealand alpine grasshoppers used for mandible analysis.

| Population | Elevation (m.a.s.l.) | Species | Sex | Collection Month / Year | Sample Size |
| --- | --- | --- | --- | --- | --- |
| Mount Hutt  (-43.5118, 171.5492) | 1450 | *Sigaus nivalis* | F | March 2016 | 9 |
|  |  |  | M |  | 8 |
|  |  | *Sigaus nitidus* | F |  | 13 |
|  |  |  | M |  | 9 |
|  |  | *Sigaus australis* | F |  | 4 |
|  |  |  | M |  | 5 |
| Craigieburn Range  (-43.125750, 171.686239) | 1480 | *S. nivalis* | F | February 2021 | 10 |
|  |  |  | M |  | 11 |
|  |  | *S. nitidus* | F |  | 7 |
|  |  |  | M |  | 12 |
|  |  | *S. australis* | F |  | 10 |
|  |  |  | M |  | 6 |
| Fox Peak  (-43.8530, 170.8077) | 1600 | *S. nivalis* | F | March 2016 | 1 |
|  |  |  | M |  | 5 |
|  |  | *S. nitidus* | F |  | 2 |
|  |  |  | M |  | 3 |
|  |  | *S. australis* | F |  | 4 |
|  |  |  | M |  | 5 |

**Table S2** Collected sites (population, geolocation, elevation), year, and sex of the three species of New Zealand alpine grasshoppers. and method used for gut content analysis (DNA metabarcoding or Microhistology).

| **Population**  **(approximate geolocation)** | **Elevation  (m.a.s.l)** | **Collection Months /**  **Year** | **Species** | **Sex** | **Sample  Size** | **Method** |
| --- | --- | --- | --- | --- | --- | --- |
| Mount Hutt  (-43.495870,  71.539220) | 1600 | February  2022 | *Sigaus nivalis* | F | 2 | DNA metabarcoding |
|  |  |  |  | M | 2 |  |
|  |  |  | *Sigaus nitidus* | F | 2 |  |
|  |  |  |  | M | 2 |  |
|  |  |  | *Sigaus australis* | F | 2 |  |
|  |  |  |  | M | 2 |  |
| Craigieburn Range  (-43.124268,  171.683578) | 1600 | February  2021 | *S. nivalis* | F | 1 | DNA metabarcoding |
|  |  |  | *S. nitidus* | F | 1 |  |
|  |  |  | *S. australis* | F | 1 |  |
|  | 1200–1800 | December 1968;  December–March 1969;  January 1970 | *S. nivalis* | F | 213 | Microhistology |
|  |  |  |  | M | 177 |  |
|  |  |  | *S. nitidus* | F | 315 |  |
|  |  |  |  | M | 266 |  |
|  |  |  | *S. australis* | F | 234 |  |
|  |  |  |  | M | 136 |  |
| Foggy Peak  (-43.2940146,  171.744291) | 1050 | February 2023 | *S. nivalis* | F | 2 | DNA metabarcoding |
|  |  |  |  | M | 2 |  |
|  |  |  | *S. nitidus* | F | 3 |  |
|  |  |  |  | M | 2 |  |
|  |  |  | *S. australis* | F | 3 |  |
|  |  |  |  | M | 1 |  |

**Table S3** List of plant species identified from the crop and gut contents of three Sigaus grasshopper species using microhistological epidermal analysis. Specimens were collected between 1968 and 1970. The taxonomic names in brackets are previously used names.

| **Structural types** | **Family** | **Genus/Species** |
| --- | --- | --- |
| Monocot | Asteliaceae | *Astelia nervosa* |
| Monocot | Cyperaceae | *Carex minor* (*Uncinia angustifolia*) |
| Monocot | Cyperaceae | *Carex horizontalis* (*Ucinia caespitosa*) |
| Monocot | Juncaceae | *Luzula pumila* |
| Monocot | Juncaceae | *Luzula rufa* |
| Monocot | Juncaceae | *Luzula traversii* |
| Monocot | Juncaceae | *Luzula* spp |
| Monocot | Juncaceae | *Marsippospermum gracile* (*Rostkovia gracilis*) |
| Monocot | Poaceae | *Agrostis dyeri* |
| Monocot | Poaceae | *Connorochloa tenuis* (*Agropyron scabrum*) |
| Monocot | Poaceae | *Anthoxanthum odoratum* |
| Monocot | Poaceae | *Chionochloa crassiuscula* |
| Monocot | Poaceae | *Chionochloa flavescens* |
| Monocot | Poaceae | *Chionochloa oreophila* |
| Monocot | Poaceae | *Chionochloa pallens* |
| Monocot | Poaceae | *Pentapogon avenoides* (*Deyeuxia avenoides*) |
| Monocot | Poaceae | *Rytidosperma pumilum* (*Erythranthera pumila*) |
| Monocot | Poaceae | *Festuca rubra* |
| Monocot | Poaceae | *Festuca novae-zelandiae* |
| Monocot | Poaceae | *Anthoxanthum recurvatum* (*Hierochloe fraseri*) |
| Monocot | Poaceae | *Lachnagrostis forsteri* |
| Monocot | Poaceae | *Zotovia colensoi* (*Microlaena colensoi*) |
| Monocot | Poaceae | *Rytidosperma setifolium* (*Notodanthonia setifolia*) |
| Monocot | Poaceae | *Poa colensoi* |
| Monocot | Poaceae | *Poa kirkii* (*Poa mackayi*) |
| Monocot | Poaceae | *Poa buchananii* (*Poa sclerophylla*) |
| Monocot | Poaceae | *Koeleria youngii* (*Trisetum youngii*) |
| Dicot | Apiaceae | *Aciphylla squarrosa* |
| Dicot | Apiaceae | *Aciphylla monroi* |
| Dicot | Apiaceae | *Anisotome aromatica* |
| Dicot | Apiaceae | *Anisotome filifolia* |
| Dicot | Apiaceae | *Chaerophyllum colensoi* (*Oreomyrrhis colensoi*) |
| Dicot | Asteraceae | *Brachyscome sinclairii* |
| Dicot | Asteraceae | *Celmisia discolor* |
| Dicot | Asteraceae | *Celmisia dubia* |
| Dicot | Asteraceae | *Celmisia durietzii* |
| Dicot | Asteraceae | *Celmisia hieraciifolia var. gracilis* |
| Dicot | Asteraceae | *Celmisia laricifolia* |
| Dicot | Asteraceae | *Celmisia lyallii* |
| Dicot | Asteraceae | *Celmisia sessiliflora* |
| Dicot | Asteraceae | *Celmisia spectabilis* |
| Dicot | Asteraceae | *Celmisia viscosa* |
| Dicot | Asteraceae | *Leptinella atrata* (*Cotula atrata*) |
| Dicot | Asteraceae | *Leptinella pyrethrifolia* (*Cotula pyrethrifolia*) |
| Dicot | Asteraceae | *Euchiton traversii* (*Gnaphalium traversii*) |
| Dicot | Asteraceae | *Haastia sinclairii* |
| Dicot | Asteraceae | *Anaphalioides bellidioides* (*Helichrysum bellidiodes*) |
| Dicot | Asteraceae | *Leucogenes grandiceps* |
| Dicot | Asteraceae | *Raoulia bryoides* |
| Dicot | Asteraceae | *Raoulia grandiflora* |
| Dicot | Asteraceae | *Raoulia subsericea* |
| Dicot | Asteraceae | *Raoulia* hybrid spp |
| Dicot | Asteraceae | *Brachyglottis lagopus* (*Senecio lagopus*) |
| Dicot | Asteraceae | *Dolichoglottis scorzoneroides* (*Senecio scorzoneroides*) |
| Dicot | Asteraceae | *Taraxacum gilliesii* (*Taraxicum magellanism*) |
| Dicot | Brassicaceae | *Cardamine* spp |
| Dicot | Campanulaceae | *Lobelia angulata* (*Pratia angulata*) |
| Dicot | Campanulaceae | *Wahlenbergia albomarginata* |
| Dicot | Caryophyllaceae | *Colobanthus acicularis* |
| Dicot | Caryophyllaceae | *Stellaria gracilenta* |
| Dicot | Caryophyllaceae | *Stellaria roughii* |
| Dicot | Ericaceae | *Styphelia nesophila (Cyathodes fraseri)* |
| Dicot | Ericaceae | *Dracophyllum pronum* |
| Dicot | Ericaceae | *Gaultheria depressa* |
| Dicot | Gentianaceae | *Gentianella corymbifera* |
| Dicot | Geraniaceae | *Geranium microphyllum* |
| Dicot | Onagraceae | *Epilobium crassum* |
| Dicot | Onagraceae | *Epilobium pedunculare* |
| Dicot | Onagraceae | *Epilobium chlorifolium (Epilobium perplexum)* |
| Dicot | Onagraceae | *Epilobium pycnostachyum* |
| Dicot | Onagraceae | *Epilobium* spp |
| Dicot | Orobanchaceae | *Euphrasia* spp |
| Dicot | Pinaceae | *Pinus* spp |
| Dicot | Pittosporaceae | *Pittosporum rigidum (Pittosporum crassicaule)* |
| Dicot | Plantaginaceae | *Veronica cheesemanii (Hebe cheesemanii)* |
| Dicot | Plantaginaceae | *Veronica epacridea (Hebe epacridea)* |
| Dicot | Plantaginaceae | *Veronica lycopodioides (Hebe lycopodioides)* |
| Dicot | Plantaginaceae | *Veronica pinguifolia (Hebe pinguifolia)* |
| Dicot | Plantaginaceae | *Ourisia caespitosa* |
| Dicot | Plantaginaceae | *Ourisia sessilifolia* |
| Dicot | Plantaginaceae | *Plantago novae-zelandiae* |
| Dicot | Plantaginaceae | *Veronica pulvinaris (Pygmaea pulvinaris)* |
| Dicot | Polygonaceae | *Muehlenbeckia axillaris* |
| Dicot | Polygonaceae | *Rumex acetosella* |
| Dicot | Primulaceae | *Myrsine nummularia* |
| Dicot | Ranunculaceae | *Ranunculus ensyii* |
| Dicot | Ranunculaceae | *Ranuculus haastii* |
| Dicot | Ranunculaceae | *Ranunculus insignis* |
| Dicot | Ranunculaceae | *Ranunculus trichophyllus* |
| Dicot | Rosaceae | *Acaena* spp |
| Dicot | Rosaceae | *Geum uniflorum* |
| Dicot | Rubiaceae | *Coprosma cheesemanii* |
| Dicot | Rubiaceae | *Coprosma pseudocuneata* |
| Dicot | Rubiaceae | *Coprosma pumila* |
| Dicot | Rubiaceae | *Asperula perpusilla (Galium perpusillum)* |
| Dicot | Stylidiaceae | *Forstera tenella* |
| Dicot | Stylidiaceae | *Phyllachne colensoi* |
| Dicot | Thymelaeaceae | *Kelleria dieffenbachii (Drapetes dieffenbachii)* |
| Dicot | Thymelaeaceae | *Pimelea prostrata* |
| Dicot | Violaceae | *Viola cunninghamii* |
| Flower | Monocot | Unidentified |
| Flower | Dicot | Unidentified, *Anisotome aromatica* |
| Fruit | Dicot | *Gaultheria depressa* |
| Fern | Blechnaceae | *Blechnum penna-marina* |
| Fern | Dryopteridaceae | *Polystichum cystostegia* |
| Fern | Hymenophyllaceae | *Hymenophyllum villosum* |
| Moss | Lycopodiaceae | *Huperzia australiana* (*Lycopodium australianum*) |
| Moss | Lycopodiaceae | *Austrolycopodium fastigiatum* (*Lycopodium fastigiatum*) |
| Moss | Polytrichaceae | *Polytrichum juniperinum* |

**Table S4** Plant chloroplast rbcL and trnL sequence reads from PCR amplicons of gut contents of 28 New Zealand alpine grasshoppers collected at Mount Hutt, Broken River and Foggy Peak after filtering, denoising, merging (for rbcL) and removing chimeric sequences.

|  | Population | Species | Sex | ID | Total | Filtered | Denoised | Merged | Chimera  removed |
| --- | --- | --- | --- | --- | --- | --- | --- | --- | --- |
| *rbc*L | Mount Hutt | *Sigaus nivalis* | F | nvF1 | 121659 | 107782 | 107613 | 107018 | 101019 |
|  |  |  |  | nvF2 | 111538 | 98149 | 97848 | 97173 | 91994 |
|  |  |  | M | nvM1 | 114520 | 100083 | 99437 | 93596 | 89627 |
|  |  |  |  | nvM2 | 129647 | 113916 | 113409 | 112359 | 107984 |
|  |  | *Sigaus nitidus* | F | ntF1 | 132535 | 115912 | 115368 | 112833 | 108899 |
|  |  |  |  | ntF2 | 136575 | 112917 | 112357 | 94280 | 91938 |
|  |  |  | M | ntM1 | 116057 | 101512 | 101297 | 100682 | 93457 |
|  |  |  |  | ntM2 | 136358 | 119397 | 119105 | 118436 | 111997 |
|  |  | *Sigaus australis* | F | auF1 | 111371 | 98703 | 98544 | 98138 | 92372 |
|  |  |  |  | auF2 | 133630 | 117499 | 116887 | 115376 | 105046 |
|  |  |  | M | auM1 | 128495 | 112542 | 112393 | 111404 | 100375 |
|  |  |  |  | auM2 | 124271 | 109785 | 109497 | 108626 | 103223 |
| *trn*L | Mount Hutt | *Sigaus nivalis* | F | nvF1 | 271040 | 251376 | 250476 |  | 236876 |
|  |  |  |  | nvF2 | 263601 | 237591 | 235648 |  | 225936 |
|  |  |  | M | nvM1 | 120809 | 108674 | 104162 |  | 96099 |
|  |  |  |  | nvM2 | 255380 | 232944 | 228681 |  | 223234 |
|  |  | *Sigaus nitidus* | F | ntF1 | 251844 | 235576 | 233588 |  | 230294 |
|  |  |  |  | ntF2 | 268206 | 246339 | 242597 |  | 227940 |
|  |  |  | M | ntM1 | 282425 | 245567 | 244647 |  | 242455 |
|  |  |  |  | ntM2 | 274978 | 239063 | 238547 |  | 233218 |
|  |  | *Sigaus australis* | F | auF1 | 265134 | 247928 | 246823 |  | 242862 |
|  |  |  |  | auF2 | 280379 | 256824 | 254325 |  | 245331 |
|  |  |  | M | auM1 | 250361 | 223558 | 222754 |  | 217230 |
|  |  |  |  | auM2 | 268475 | 250554 | 249323 |  | 242787 |
|  | Broken River | *S. nivalis* | F | nvF3 | 286684 | 266117 | 264124 |  | 255437 |
|  |  | *S. nitidus* | F | ntF3 | 254821 | 236129 | 234110 |  | 225769 |
|  |  | *S. australis* | F | auF3 | 175107 | 157564 | 153038 |  | 144730 |
|  | Foggy Peak | *S. nivalis* | F | nvF4 | 435298 | 414868 | 413109 |  | 392280 |
|  |  |  |  | nvF5 | 102297 | 88152 | 87732 |  | 86628 |
|  |  |  | M | nvM3 | 236771 | 219948 | 219154 |  | 217459 |
|  |  |  |  | nvM4 | 29349 | 27821 | 27652 |  | 27498 |
|  |  | *S. nitidus* | F | ntF4 | 456878 | 439483 | 438437 |  | 437105 |
|  |  |  |  | ntF5 | 130632 | 111989 | 111983 |  | 111244 |
|  |  |  |  | ntF6 | 250492 | 233271 | 232155 |  | 227597 |
|  |  |  | M | ntM3 | 383179 | 369416 | 369052 |  | 357291 |
|  |  |  |  | ntM4 | 290434 | 278899 | 278867 |  | 278227 |
|  |  | *S. australis* | F | auF4 | 2117171 | 2036153 | 2035633 |  | 1947641 |
|  |  |  |  | auF5 | 105845 | 94105 | 92743 |  | 86956 |
|  |  |  |  | auF6 | 106209 | 92926 | 92274 |  | 91707 |
|  |  |  | M | auM3 | 25642 | 24137 | 24008 |  | 23313 |

**Table S5.** Number of taxa identified at family, genus and species levels in each gut content of New Zealand alpine grasshoppers using different identification markers (rbcL and trnL). Taxa of rbcL and trnL sequences were identified using a custom reference database and BLAST+ in QIIME2.

| Species | Sex | ID | *rbc*L | | | *trn*L | | |
| --- | --- | --- | --- | --- | --- | --- | --- | --- |
|  |  |  | Family | Genus | Species | Family | Genus | Species |
| *Sigaus nivalis* | F | nvF1 | 8 | 6 | 1 | 10 | 7 | 1 |
|  |  | nvF2 | 8 | 6 | 1 | 8 | 6 | 0 |
|  | M | nvM1 | 6 | 5 | 1 | 10 | 9 | 3 |
|  |  | nvM2 | 8 | 7 | 1 | 10 | 8 | 2 |
| *Sigaus nitidus* | F | ntF1 | 3 | 2 | 0 | 8 | 8 | 1 |
|  |  | ntF2 | 6 | 3 | 0 | 8 | 8 | 1 |
|  | M | ntM1 | 6 | 6 | 1 | 6 | 4 | 0 |
|  |  | ntM2 | 6 | 5 | 0 | 10 | 8 | 2 |
| *Sigaus australis* | F | auF1 | 6 | 4 | 0 | 8 | 8 | 0 |
|  |  | auF2 | 8 | 5 | 1 | 9 | 6 | 1 |
|  | M | auM1 | 9 | 8 | 1 | 9 | 8 | 2 |
|  |  | auM2 | 8 | 7 | 1 | 9 | 7 | 1 |

**Table S6.** Comparison of plant taxonomic identification from rbcL and trnL chloroplast to family, genus and species level. Shading indicates a higher taxonomic identification from rbcL or trnL of the same sample.

|  | ***rbc*L** | | | | ***trn*L** | | | |
| --- | --- | --- | --- | --- | --- | --- | --- | --- |
|  | *Sigaus nivalis* | | | | | | | |
|  | nvF1 | nvF2 | nvM1 | nvM2 | nvF1 | nvF2 | nvM1 | nvM2 |
| Family | 99.10% | 99.65% | 99.38% | 99.91% | 99.98% | 99.88% | 99.94% | 99.94% |
| Genus | 3.00% | 80.82% | 94.61% | 96.15% | 87.33% | 69.40% | 96.76% | 98.48% |
| Species | 0.02% | 0.03% | 1.29% | 0.02% | 1.34% | 0.00% | 2.96% | 0.74% |
|  | *Sigaus nitidus* | | | | | | | |
|  | ntF1 | ntF2 | ntM1 | ntM2 | ntF1 | ntF2 | ntM1 | ntM2 |
| Family | 99.68% | 97.78% | 99.91% | 99.83% | 99.98% | 99.91% | 99.98% | 100.00% |
| Genus | 2.65% | 5.47% | 96.64% | 93.05% | 5.65% | 59.60% | 98.22% | 96.11% |
| Species | 0.00% | 0.00% | 0.04% | 0.00% | 0.00% | 0.01% | 0.00% | 0.18% |
|  | *Sigaus australis* | | | | | | | |
|  | auF1 | auF2 | auM1 | auM2 | auF1 | auF2 | auM1 | auM2 |
| Family | 99.56% | 99.49% | 99.94% | 99.63% | 99.97% | 99.84% | 99.98% | 99.79% |
| Genus | 4.23% | 22.43% | 95.84% | 16.67% | 88.49% | 35.33% | 85.88% | 19.04% |
| Species | 0.00% | 0.01% | 0.04% | 0.04% | 0.00% | 0.00% | 0.26% | 1.61% |
